# Tapping into truth: an exploratory cross-sectional analysis of psychomotor symptoms and typing behaviour in an adolescent observational cohort

**DOI:** 10.1038/s44277-025-00033-0

**Published:** 2025-06-18

**Authors:** Taylor A. Braund, Debopriyo Bal, Helen Christensen, Philip J. Batterham, Bojana Vilus, Kate Maston, Mark E. Larsen, Aliza Werner-Seidler, Kit Huckvale, Alexis E. Whitton, Gabriel Tillman, Bridianne O’Dea

**Affiliations:** 1 Black Dog Institute, University of New South Wales, Sydney, NSW Australia; 2 Faculty of Medicine and Health, University of New South Wales, Sydney, NSW Australia; 3 Centre for Mental Health Research, The Australian National University, Canberra, Australia; 4 Centre for Big Data Research in Health, University of New South Wales, Sydney, NSW Australia; 5 Centre for Digital Transformation of Health, Faculty of Medicine, Dentistry and Health Sciences, The University of Melbourne, Melbourne, VIC Australia; 6 Australian Institute of Family Studies, Melbourne, VIC Australia; 7 Flinders University Institute for Mental Health and Wellbeing, Flinders University, South Australia, Australia

**Keywords:** Biomarkers, Signs and symptoms

## Abstract

Typing behaviour derived from smartphone keystroke metadata is an emerging digital phenotype that may assist in diagnosing and monitoring depressive symptoms. While psychomotor agitation and slowing have been hypothesised as depressive symptoms that may influence typing behaviour, no studies have directly tested this assumption. Here, we tested whether specific depressive symptoms were associated with various keystroke features of typing behaviour in adolescents. Adolescents from an Australian cohort study (*n* = 895) completed a typing task on their smartphones. Common features of keystroke timing (i.e., median, dwell, interval, latency, down-down time, and up-up time) and frequency (i.e., total keystrokes, backspaces, spaces, backspace ratio, and spaces ratio) were extracted. Depressive symptoms were assessed using the Patient Health Questionnaire-Adolescent version (PHQ-A). Multiple linear regression models were used to test associations between symptom items and keystroke features. Non-linear effects and moderating effects of sex were also explored. Psychomotor symptoms (i.e., PHQ-A item 8) were not associated with keystroke timing or frequency. However, higher appetite symptoms (i.e., PHQ-A item 5) were associated with faster down-down time and a greater number of total key presses. Symptoms of anhedonia (i.e., PHA item 1) showed non-linear associations with keystroke features. The results do not support a relationship between psychomotor symptoms and typing behaviour in adolescents. However, appetite-related symptoms were associated with faster and more frequent typing. Further research into the relationship between typing behaviour and mental health in young people is warranted. Clinical Trial Registry: ACTRN12619000855123

## Lay summary

We examined whether teenagers' smartphone typing behaviours could reflect specific depressive symptoms. Contrary to expectations, we found no link between typing patterns and psychomotor changes, like agitation or slowing. Interestingly, adolescents experiencing increased appetite typed faster and pressed more keys, while symptoms of anhedonia showed complex associations. These findings suggest smartphone typing patterns might offer a useful tool for understanding and monitoring particular depression symptoms in young people.

## Introduction

Keystroke dynamics, which refer to the timing and rhythm of typing behaviour, has recently emerged as a promising digital phenotype for understanding mental health conditions, particularly depression [1–3]. Keystroke data may be advantageous for providing unobtrusive and remote monitoring of depressive symptoms. However, findings from studies investigating the associations between keystroke features and depression have been mixed. Early work from Zulueta, et al. [3] using the BiAffect custom keyboard found higher depressive symptoms were associated with slower typing speed (i.e., increased average interkey delay). Using typing data collected from Mindstrong Health, Yang. et al. [4] found that higher depressive symptoms were also associated with slower typing speed (i.e., increased typing time interval) when analysed longitudinally within-person, but found no association when assessed cross-sectionally. In contrast, Braund, et al. [1] found higher levels of self-reported depressive symptoms were associated with faster typing speed and more frequent key presses when examined cross-sectionally in adolescents using a custom built smartphone application. However, these associations were weak and non-significant after the adjustments for multiple comparisons. These inconsistencies across studies highlight the complexity of the relationship between depression and keystroke dynamics, suggesting the relationship may not be uniform across different populations or contexts. Furthermore, while much of the existing research has focused on overall depressive symptom severity, it is commonly assumed that the observed relationships between keystroke features and depression are primarily caused by specific depressive symptoms [4].

Psychomotor symptoms, such as psychomotor slowing and agitation, are considered core features of depression and are commonly theorised to contribute to the subtle variations in typing behaviour [3–5]. For example, appetite dysregulation—whether through undereating or overeating—has been linked to fluctuations in energy levels and motor activity [6]. Individuals with restrictive eating behaviours, such as those seen in anorexia nervosa, often exhibit increased movement and restlessness, despite reduced caloric intake, potentially due to dopaminergic dysregulation [7, 8]. In contrast, overeating and weight gain have been associated with fatigue, reduced physical activity, and psychomotor slowing [9, 10]. These patterns suggest that appetite-related changes may extend beyond gross motor function to influence fine motor behaviours, such as typing. Similarly, psychomotor slowing could manifest as longer keystroke latencies, reflecting a decrease in cognitive and motor speed, while psychomotor agitation might result in more erratic and less consistent typing patterns, characterised by increased variability in keystroke intervals. Other depressive symptoms such as sleep disturbances and appetite changes might also influence typing behaviour. Sleep disturbances, like insomnia or hypersomnia, are prevalent in depression and can lead to cognitive impairments such as slower reaction times, reduced attention, and decreased processing speed [11–13]. These cognitive deficits could be reflected in keystroke dynamics, potentially resulting in slower typing speeds, increased errors, and more frequent pauses during typing tasks. Despite the theoretical and empirical connections between specific symptoms of depression and typing behaviour, no studies to date have directly investigated these associations.

The current study aimed to examine the associations between specific symptoms of depression and common keystroke timing and frequency features. Based on previous findings from Braund, et al. [1], it was hypothesised that greater severity of the psychomotor symptoms would be associated with faster keystrokes and more frequent keystrokes. Prior research indicates that psychomotor activity and cognitive-motor performance can often exhibit non-linear relationships with symptom severity. For example, mild sleep deprivation can temporarily increase arousal and motor speed, whereas severe sleep deprivation impairs reaction time and cognitive function [14, 15]. As such, we also included quadratic terms in our models to test whether keystroke dynamics followed a similar pattern, particularly in relation to appetite and sleep disturbances. Additional analyses explored the moderating effect of sex on these relationships.

## Materials and method

### Design

This study was a secondary analysis of baseline data from a prospective cohort study of adolescents with an embedded cRCT (i.e., The Future Proofing Study). The trial protocol [16] and baseline characteristics [17] have been published elsewhere. Ethics approvals were obtained from the University of New South Wales Human Research Ethics Committee (HC180836), the State Education Research Applications Process for the New South Wales Department of Education (SERAP2019201), and relevant Catholic Schools Dioceses across Australia.

### Setting

The Future Proofing Study was conducted in 134 secondary schools in Australia, including government and non-government (independent and Catholic) schools. Recruitment was conducted from March 2019 to March 2022. All New South Wales (NSW) government and independent secondary schools and eligible NSW Catholic secondary schools were invited to participate. Independent schools in capital cities from around Australia were also invited to participate. Written informed consent was provided from a parent or guardian and the adolescent prior to study participation. Data collection took place across three separate Year 8 cohorts (students aged 13–14 years).

### Participants

Participants (*N* = 6388) were secondary school adolescents from Year 8 who attended participating schools. All adolescents enrolled in Year 8 at each participating school were eligible to participate in the trial if they had a smartphone with iOS or Android operating system and an active phone number. All participants were invited to undertake the text typing task as part of omnibus of digital tasks (see [17]). Participants were limited to those who completed the relevant text typing task at baseline (*n* = 1167/6388, 18.9%), those who completed the typing task in the same two week period that they completed their baseline questionnaire (*n* = 918/1167, 78.7%), and those whose text responses aligned with the script (as opposed to a random body of text) and contained less than 50% gibberish (defined as non-meaningful text characterised by repeated letters or random sequences of characters, commonly referred to as key mashing; *n* = 895/918, 97.5%).

### Procedure

Participants with parental consent completed the study questionnaires via a secure web-based portal, accessible using their phone number and a one-time password sent via SMS. Following completion of baseline questionnaires, participants were instructed to download and open the Future Proofing App to complete the text typing task during class time if time was provided, and in their own time.

### Text typing task

Participants completed the text typing task through the Future Proofing App. Participants were asked to type a script of text as quickly as possible within 30 s. Scripts were randomly allocated to participants from a pool of 8 (see Supplementary Material Fig. 1 for screenshots of the typing task and Supplementary Material for typing scripts).

### Measures

#### Depressive symptoms

The Patient Health Questionnaire for Adolescents (PHQ-A) is a nine-item self-administered depression severity screening and diagnostic tool based on DSM-IV criteria for Major Depressive Disorder (MDD) validated for use in adolescents [18]. The scale assesses the frequency of occurrence of nine depression symptom criteria during the previous 2 weeks, with items rated on a 4-point scale ranging from 0 (‘Not at all’) to 3 (‘Nearly every day’). Total scale scores on the PHQ-A can range from 0–27, with higher scores reflecting more severe depressive symptoms. The internal consistency of the PHQ-A in the current study was high (*α* = 0.89; see Supplementary Material Fig. 2 for distributions of PHQ-A items and Supplementary Material Table 1 for means and standard deviations of PHQ-A items). Correlations between individual PHQ-A items were mostly moderate, ranging from *r* = 0.31–0.69 (see Supplementary Material Table 2 for all correlations).

#### Anxiety symptoms

The Children’s Anxiety Scale Short-Form (CAS-8) is an 8-item brief measure of anxiety for children and adolescents, based on the Spence Children’s Anxiety Scale [19]. The CAS-8 incorporates questions assessing generalized anxiety and social anxiety. Respondents rate the degree to which they experience each symptom on a 4-point frequency scale, ranging from 0 “Never” to 3 “Always.” The CAS-8 has demonstrated good reliability and provides population-level, standardized norms, with a range of 0–24 (higher score indicates greater anxiety; [20, 21]). The internal consistency of the CAS-8 in the current study was high (*α* = 0.94).

#### Insomnia

The Insomnia Severity Index (ISI) is a psychometrically sound, seven-item self-report measure of insomnia symptoms over the previous 2 weeks [22]. Responses are reported on a Likert scale ranging from 0 (‘Not at all’) to 4 (‘Very’), producing total scores of 0–28. The ISI was designed for use in adults but has been widely administered to, and validated in, adolescent samples [23, 24]. The internal consistency of the ISI in the current study was high (*α* = 0.88).

#### Screen for disordered eating questionnaire

The Screen for Disordered Eating (SDE; [25] is a 5-item scale recently developed to screen for eating disorders. Respondents indicate whether they have experienced five symptoms of disordered eating using a dichotomous scale (‘Yes’ or ‘No’). An individual is screened as positive if they endorse two or more items. This measure has demonstrated good discriminative accuracy in primary care [25]. The internal consistency of the SDE in the current study was moderate (*KR-20* = 0.67).

### Preprocessing and feature extraction

Keystroke timing features of median dwell (i.e., the time interval between a key press and release of the same key), latency (i.e., the time interval between a key press of a keystroke and key release of the following key), interval (i.e., the time interval between a key release of a keystroke and key press of the following keystroke), up-up time (i.e., the time interval between key release of a keystroke and key release of the following keystroke), and down-down time (i.e., the time interval between key press of a keystroke and key press of the following keystroke) were extracted for each participant. Keystroke frequency features included total keystrokes, total backspace, total spaces, as well as the proportion of total backspace and total spaces [26]. Formulas and descriptions of all keystroke features extracted are provided in the Supplementary Material Table 3. Distributions of features before and after transformation are provided in in the Supplementary Material Fig. 3.

### Statistical analysis

To test whether any of the individual PHQ-A symptom items were associated with keystroke features, we used mixed-effect linear regression models with cluster robust standard errors. The CR2 estimator was used for the cluster robust standard errors, which adjusts for within-cluster correlation and small sample bias. Keystroke features were included as the dependent variables, with a separate model estimated for each keystroke feature. All individual PHQ-A items were included simultaneously in each of these models as independent variables. Potential confounds were also adjusted for in each model, including sex, age, handedness for typing, location, language spoken at home, and duration between mental health assessment and typing task. Text typing script category was included as a random effect. To quantify the proportion of variance attributable to differences in text typing script, the intraclass correlation coefficient (ICC) was calculated for each model. As four out of the five keystroke timing features (i.e., latency, interval, up-up time, and down-down time) were highly correlated (i.e., >0.9; see Supplementary Material Tables 4 and 5 for correlations between keystroke features), only down-down time (also known as ‘interkey delay’) was analysed as it is the most common keystroke timing feature reported in the literature [2, 3, 27]. Total keystrokes and total spaces were also highly correlated (i.e., >0.9), so only total keystrokes was analysed. Positively skewed keystroke features (i.e., down-down time, total backspaces, and backspace ratio) were transformed before analysis to meet assumptions of constant residual variance [28]. For methods of transformation and distributions of skewed data before and after transformation, see Supplementary Material. To assess non-linearity in the relationships between PHQ-A items and keystroke features, we compared nested models with and without quadratic terms. Quadratic terms were modelled using orthogonal polynomials to reduce collinearity. Finally, to test whether any of the significant relationships were moderated by sex, we compared nested models that included interaction terms between sex and significant PHQ-A items, where sex was reduced to male and female categories. The statistical significance of improvements in model fit were evaluated using the F-statistic from a Wald test. A significant F-statistic indicated that the extended model provides a statistically better fit to the data. To assess the robustness of the relationships identified between keystroke features and PHQ-A items, alternative symptom measures from other validated questionnaires (e.g., SDE, ISI, and CAS-8) were similarly analysed using multiple linear regression models with robust standard errors. Predicted values from models with significant predicters were plotted, with log transformed outcome variables back transformed for interpretability. Duan [29] smearing estimator was applied as a non-parametric method to correct for the retransformation bias introduced when back-transforming log-transformed predictions in the regression models. This approach ensures unbiased estimates on the original scale by adjusting for the skewness introduced by the log transformation. Diagnostic plots for all models are provided in the Supplementary Material Figs. 4–15. As diagnostic plots identified potential outlier observations, we conducted sensitivity analysis using robust linear mixed-effects regression to down-weight the influence of outlier observations (see Supplementary Material Tables 6–17). Robust linear mixed-effects models down-weight influential observations by iteratively adjusting observation-level weights during parameter estimation. This approach preserves the data, limits outlier influence on both fixed and random effects, and often results in larger (more conservative) standard errors compared to traditional methods that remove such observations. Any changes observed in the direction or significance of our main effects from the sensitivity analysis are reported in the results. Because robust models do not directly provide p-values, p-values were calculated from robust t-values using Satterthwaite-approximated degrees of freedom. This approach provides an approximate significance test for exploratory purposes, acknowledging that robust models do not inherently support p-value computation. Sensitivity analysis was also completed to assess whether normalising days between assessment and typing task in models impacted results. These additional analyses demonstrated minimal impact on the overall model parameters and no change in primary conclusions. Thus, the original metric was retained for interpretability.

All analyses were performed using R 4.4.0 [30]. Mixed-effect linear models were implemented using the ‘lme4’ [31] and ‘lmerTest’ [32] R packages. Robust standard errors were implemented using the ‘clubSandwich’ [33] package in R. ICC was calculated and model diagnostic plots were generated using the ‘performance’ [34] package in R. Robust linear mixed-effects models were implemented using the ‘robustlmm’ R package [35]. Figure plots were generated using the ‘ggplot2’ [36] and ‘ggeffects’ [37] packages in R.

## Results

### Participants

Participant characteristics are presented in Table 1. The mean age was 13.82 years (*SD* = 0.56), the mean PHQ-A score was 8.01 (*SD* = 6.57), and 64.7% (*n* = 579) were female. Latency had the longest duration (*M* = 301.69; *SD* = 74.82) and dwell had the shortest duration (*M* = 78.98; *SD* = 13.70) among keystroke timing features, and the mean total keystrokes was 96.86 (*SD* = 31.24). Most participants self-reported that they typed with both hands (68.7%; *n* = 615) and 21.0% (*n* = 188) completed the task on an iPhone.

**Table 1:** Demographic and clinical characteristics of participants.

	Total sample	
	*N* = 895	
	*M*	*SD*
Age (years)	13.82	0.56
Depressive symptoms (PHQ-A)	8.01	6.57
Anxiety symptoms (CAS-8)	9.31	5.65
Insomnia symptoms (ISI)	7.77	5.90
Disordered eating symptoms (SDE)	1.68	1.49
Keystroke timing features (Median, ms)		
Dwell	78.98	13.70
Latency	301.69	74.82
Interval	146.92	65.94
Down-down time	221.03	69.40
Up-up time	224.94	69.37
Keystroke frequency features (total)		
Keystrokes	96.86	31.24
Spaces	16.40	6.34
Backspaces	7.58	6.54
Keystroke Frequency Features (proportion)		
Spaces	16.81	3.15
Backspaces	8.14	7.15
Duration between mental health assessment and typing days (days)	1.95	3.26
	*N*	%
Sex		
Female	579	64.7
Male	308	34.4
Not sure	4	0.4
Other	4	0.4
Location		
Major cities	666	74.4
Inner regional	200	22.3
Outer regional	29	3.2
Language spoken at home		
English	834	93.2
Other	61	6.8
Typing hand		
Both	615	68.7
Right	57	6.4
Left	7	0.8
Not reported	216	24.1
Device		
iPhone	188	21.0
Android	49	5.5
Not reported	658	73.5
Text typing script		
“For a minute or two…”	109	12.2
“There was a table set…”	117	13.1
“So much I’ll tell you…”	113	12.6
“Then the sound of…”	115	12.8
“They were friends in…”	106	11.8
“The winter was very…”	119	13.3
“A little girl, radiant…”	101	11.3
“But they by no means…”	115	12.8

### PHQ-A symptoms associated with keystroke timing features

**Fig. 1:**
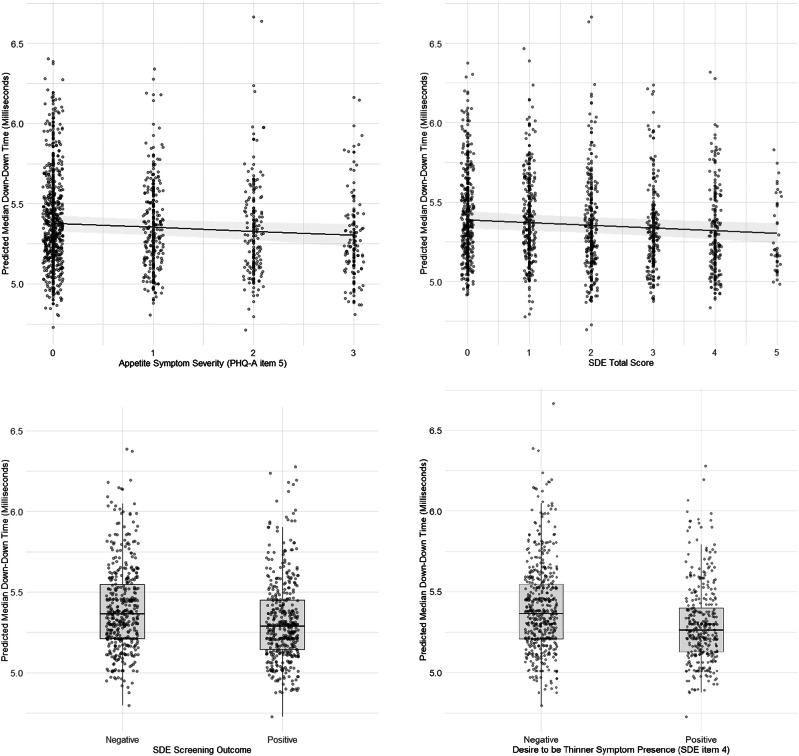
Predictor effect plots showing associations between eating-related symptom severity and predicted median down-down time. Shaded regions represent 95% confidence intervals, and points represent individual data.

For all results from final fitted models, see Supplementary Material Tables 6–17. ICC’s ranged from 0.064 to 0.068 for keystroke timing models, indicating minimal variance attributable to differences in the typing script. Psychomotor symptoms (i.e., PHQ-A item 8) were not significantly associated with down-down time (*b* = −0.01, *SE* = 0.01, *95% CI* [−0.04, 0.02], *t* = −0.99, *p* = 0.358). However, higher appetite symptoms (i.e., PHQ-A item 5) were associated with a faster down-down time (*b* = −0.02, *SE* = 0.01, *95% CI* [−0.05, 0.00], *t* = −2.41, *p* = 0.048; see Fig. 1). No PHQ-A items were significantly associated with dwell (all *p* > 0.05). The inclusion of quadratic terms or interaction terms between sex and PHQ-A items did not improve the model fit (all *p* > 0.05).

### Exploratory analysis of symptom scales assessing disordered eating, sleep, and anxiety

Exploratory analyses using models with identical covariates were conducted to test whether relationships between down-down time and symptoms of disordered eating, sleep, and anxiety were observed from the SDE, ISI, and CAS-8 questionnaires. For disordered eating, higher disordered eating total score was associated with faster down-down time (*b* = −0.02, *SE* = 0.01, *95% CI* [−0.03, −0.00], *t* = −2.39, *p* = 0.049). Similarly, screening positive for disordered eating was associated with faster down-down time (*b* = −0.05, *SE* = 0.02, *95% CI* [−0.08, −0.02], *t* = −3.76, *p* = 0.007). When comparing nested models to assess the contribution of non-linear terms, the inclusion of a quadratic term for disordered eating total score did not significantly improve the model fit (*F*(1, 7) = 0.26, *p* = 0.628). When testing a model with individual items, higher scores on SDE item 4 (*b* = −0.06, *SE* = 0.02, *95% CI* [−0.12, −0.00], *t* = −2.46, *p* = 0.045) was associated with faster down-down time. Introducing an interaction term between SDE item 4 and sex did not improve the model (*F*(1, 6) = 0.11, *p* = 0.750).

For insomnia, ISI total score was not associated with down-down time (*b* = 0.00, *SE* = 0.00, *95% CI* [0.00, 0.00], *t* = −1.24, *p* = 0.256). Including a non-linear term (*F*(1, 6) = 2.46, *p* = 0.166) or sex interaction term (*F*(1, 7) = 1.98, *p* = 0.205) did not improve the model fit.

For anxiety, CAS-8 total score was not associated with down-down time (*b* = 0.00, *SE* = 0.00, *95% CI* [0.00, 0.00], *t* = −0.65, *p* = 0.539). Including a non-linear term (*F*(1,76) = 0.52, *p* = 0.494) or sex interaction term (*F*(1, 7) = 2.71, *p* = 0.147) did not improve the model fit.

### Significant covariates in keystroke timing models

Across both the main and sensitivity analyses, three covariates were consistently significant across all keystroke timing models: sex, handedness, and location. Male participants typed slower than female participants (b range = 0.10–0.12, all *p* < 0.001); right handed typists were slower than those using both hands (b range = 0.15–0.16, *p* range = 0.010–0.018), and participants residing in major cities typed faster than those in inner regional areas (b range = −0.08 to ., p range = 0.012–0.021). Greater time between assessment and typing task was associated with slower typing in some models (b range = 0.004–0.005, p range = 0.036–0.063), but were not significant in any sensitivity analysis models. Covariates age and English spoken at home showed no association with typing speed.

### PHQ-A symptoms associated with keystroke frequency features

ICC’s ranged from 0.066 to 0.075 for keystroke frequency models, indicating minimal variance attributable to differences in the typing script. Higher scores on PHQ-A item 1 (i.e., little interest or pleasure in doing things) was negatively associated with a total keystrokes (*b* = −2.90, *SE* = 0.86, *95% CI* [−4.93, −0.87], *t* = −3.39, *p* = 0.012) and item 5 was positively associated with total keystrokes (*b* = 4.56, *SE* = 0.86, *95% CI* [2.52, 6.59], *t* = 5.32, *p* < 0.001). PHQ-A item 8 was not significantly associated with total keystrokes (*B* = 0.49, *SE* = 0.85, *95% CI* [−1.53, 2.51], *t* = 0.57, *p* = 0.584). No PHQ-A items were associated with total backspace, backspace ratio, or space ratio (all *p* > 0.05).

**Fig. 2:**
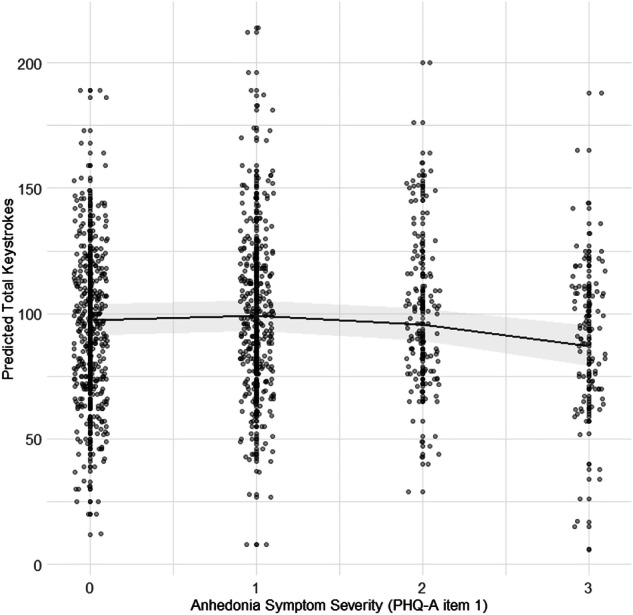
Predictor effect plots showing associations between anhedonia symptom severity and predicted total keystrokes. Shaded regions represent 95% confidence intervals, and points represent individual data.

When comparing nested models to assess the contribution of non-linear terms, the inclusion of a quadratic term for PHQ-A item 1 significantly improved the model fit (*F*(1, 7) = 8.15, *p* = 0.025). The coefficient of the quadratic term for PHQ-A item 1 was negative (*b* = −70.16, *SE* = 24.58, *95% CI* [−128.35, −11.96], *t* = −2.85, *p* = 0.025; see Fig. 2), suggesting that at lower symptom levels, increases in PHQ-A item 1 scores were associated with a slight rise in typing frequency, but as symptoms became more severe, typing frequency declined at an accelerating rate. However, after conducting a sensitivity analysis using robust regression methods to account for influential observations, the association was attenuated and no longer statistically significant (*t* = −1.75, *p* = 0.081). While the effect estimate remained in the same direction, the reduced magnitude and loss of significance warrant cautious interpretation. The inclusion of a quadratic term for PHQ-A item 5 did not significantly improve the model fit (*F*(1, 7) = 1.12, *p* = 0.325).

When comparing nested models to assess the potential moderating effect of sex, including an interaction term between sex and the quadratic term for PHQ-A item 1 did not significantly improve the model fit (*F*(1, 7) = 1.65, *p* = 0.241).

### Exploratory analysis of symptom scales assessing disordered eating, sleep, and anxiety

**Fig. 3:**
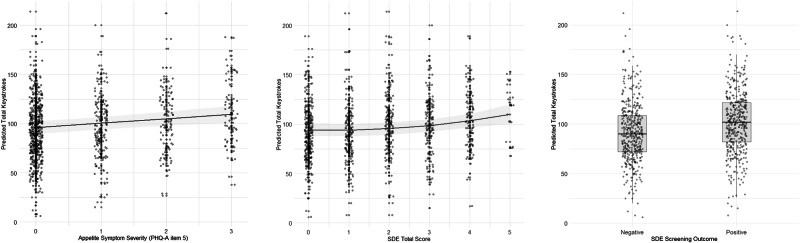
Predictor effect plots showing associations between eating-related symptom severity and predicted total keystrokes. Shaded regions represent 95% confidence intervals, and points represent individual data.

Exploratory analyses using models with identical covariates were conducted to test whether relationships between total keystrokes and symptoms of disordered eating, sleep, and anxiety were observed from the SDE, ISI, and CAS-8 questionnaires. For disordered eating, higher disordered eating total scores were associated with higher total keystrokes (*B* = 2.36, *SE* = 0.87, *95% CI* [0.29, 4.44], *t* = 2.70, *p* = 0.031; see Fig. 3). Similarly, a positive screen for disordered eating was associated with higher total keystrokes (*B* = 6.22, *SE* = 2.13, *95% CI* [1.18, 11.26], *t* = 2.92, *p* = 0.023). When comparing nested models to assess the contribution of non-linear terms, the inclusion of a quadratic term for disordered eating total score significantly improved the model fit (*F*(1, 7) = 6.14, *p* = 0.044).

The coefficient of the quadratic term for disordered eating total score was positive (*b* = 50.34, *SE* = 20.31, *95% CI* [1.82, 98.87], *t* = 2.48, *p* = 0.044), suggesting that at lower symptom levels, SDE scores were associated with a slight decline in typing frequency, but as symptoms became more severe, typing frequency increased at an accelerating rate. When comparing nested models to assess the potential moderating effect of sex, including an interaction term between sex and the quadratic term for SDE total score did not significantly improve the model fit (*F*(1, 6) = 0.06, *p* = 0.814). When testing a model with individual SDE items, no items were associated with total keystrokes (all *p* > 0.05).

For insomnia, ISI total score was not associated with total keystrokes (*b* = 0.39, *SE* = 0.20, *95% CI* [−0.08, 0.85], *t* = 1.99, *p* = 0.089). Including a non-linear term (*F*(1, 6) = 1.22, *p* = 0.309) or sex interaction term (*F*(1, 7) = 4.02, *p* = 0.087) did not improve the model fit.

For anxiety, CAS-8 total score was not associated with total keystrokes (*b* = 0.12, *SE* = 0.17, *95% CI* [-0.28, 0.52], *t* = 0.73, *p* = 0.491). Including a non-linear term (*F*(1, 7) = 0.07, *p* = 0.799) or sex interaction term (*F*(1, 7) = 2.27, *p* = 0.178) did not improve the model fit.

### Significant covariates in keystroke frequency models

Across both the main and sensitivity analyses, three covariates were consistently significant across all keystroke frequency models: sex, handedness, and location. Male participants typed less frequently than female participants (*b* range = –12.89 to –15.19, all *p* < 0.001); right-handed typists typed less frequently than those using both hands (*b* range = –13.99 to –14.88, *p* range = <0.001–0.008); and participants residing in major cities typed more frequently than those in inner regional areas (*b* range = 7.49–7.94, *p* range = 0.016–0.025). All other covariates—including age, English spoken at home, and days between assessment and typing task—showed no consistent association with keystroke frequency.

## Discussion

This study investigated associations between specific symptoms of depression and common keystroke timing and frequency features in adolescents. While no significant associations were found between the psychomotor item (i.e., PHQ-A item 8) and typing behaviour, higher appetite symptoms (i.e., PHQ-A item 5) were associated with faster down-down time and a greater number of total key presses. Furthermore, anhedonia symptoms (i.e., PHQ-A item 1) showed non-linear associations with keystroke features.

The findings of this study suggest that disordered eating may have measurable effects on the motor functions of adolescents, as reflected in typing behaviour. One possible explanation for this association is that changes in appetite might be accompanied by fluctuations in energy levels and overall physical activity [6], which could influence typing behaviour. Faster and more frequent keystrokes may also signal underlying disordered eating illnesses such as anorexia nervosa, given that 80% of anorexia nervosa patients reported increased urge for movement or physical restlessness [7]. Future research should incorporate other data that may be indicative of disordered eating such as body weight or weight loss over time. The link between appetite changes and keystroke behaviour may also reflect underlying physiological or neurobiological mechanisms associated with depression. Appetite dysregulation in depression has been linked to alterations in the hypothalamic-pituitary-adrenal (HPA) axis and changes in neurotransmitter systems, such as serotonin, which play crucial roles in both mood regulation and motor control [6, 8, 9]. These biological changes could manifest in increased motor activity or restlessness, as observed in the faster down-down time times and more frequent key presses.

Beyond the linear associations, the current findings also suggest non-linear relationships between depressive symptoms and typing behaviour. Adolescents typing frequency notably decreased as anhedonia symptoms (PHQ-A item 1) became more severe, while typing frequency increased as disordered eating symptoms became more severe. These patterns may reflect underlying mechanisms that become increasingly pronounced at higher symptom severity, such as heightened psychomotor slowing and motivational deficits in severe anhedonia, or intensified restlessness and behavioural activation at higher levels of disordered eating symptoms [38, 39]. At extreme symptom levels, these mechanisms could drive rapid, exponential changes in motor behaviour, resulting in distinct typing patterns. Future research should directly investigate these potential threshold effects to clarify whether exponential shifts in typing behaviour correspond to meaningful clinical deterioration or distinct symptom profiles.

Our study identified consistent effects of sex, handedness, and geographic location on keystroke dynamics across both timing and frequency models. Specifically, male participants and right-handed typists exhibited slower typing speeds and lower typing frequency compared to female and left-handed individuals, respectively. In contrast, residents of major urban areas demonstrated faster and more frequent typing behaviours than those in less urbanised regions. These findings are consistent with research indicating that males may exhibit slower typing due to sex differences in fine motor speed and psychomotor processing efficiency, with females outperforming males on tasks requiring rapid coordinated finger movements [40, 41], while urban residents’ faster typing likely reflects greater exposure to digital technology and higher digital literacy driven by socioeconomic and infrastructural advantages [42]. Notably, the associations between depressive symptoms and keystroke features remained significant even after adjusting for these covariates, highlighting the robustness of this relationship. However, the underlying mechanisms driving these demographic differences, such as motor control variations or socioeconomic factors, warrant further investigation to fully elucidate their impact on keystroke dynamics.

There are several limitations in the current study. Features of typing behaviour were extracted from data collected from a text typing task at a single timepoint within two weeks of the depression symptoms. Our study used a brief 30‐second text copying task rather than naturalistic, everyday typing data. Although copying tasks are a recognised proxy for assessing fine motor function—comparable to writing or drawing tasks used in clinical assessments [43] —this approach may not fully capture the nuances of natural smartphone use or subtle psychomotor variations. Additionally, the speed‐focused instructions could have induced a speed–accuracy trade-off [44], further limiting the interpretation of psychomotor effects. The cross-sectional design prevents examination of within-person changes in typing behaviour over time. Continuous collection of typing data over an extended period could provide a more detailed and dynamic understanding of how typing behaviour fluctuates with depressive symptoms, potentially capturing more nuanced short-term and long-term changes [45, 46]. The study sample consisted of adolescents, which may limit the generalisability of the findings to other age groups. Different age groups might exhibit different patterns of typing behaviour in relation to depressive symptoms [3, 4]. The cross-sectional design of the study also limits the ability to make causal inferences about the relationship between depressive symptoms and typing behaviour. Depression symptoms were assessed using self-reported measures and may not generalise to the broader literature, which has mostly assessed depression using clinician rated scales (for a review of studies, see [47]). The psychomotor symptom item on the PHQ has not been directly validated against objective measures of psychomotor function, such as gait speed, reaction time, or actigraphy. While broader research links psychomotor functioning in depression to physical performance metrics [39, 48], future studies should assess whether this specific PHQ-A item reliably captures objective motor changes. Finally, several items in the PHQ-A are double-barrelled, meaning they ask about more than one symptom at a time (e.g., “feeling down or irritable”). This complicate interpretation, as it is unclear whether participants are responding to one or both aspects of the question and potentially affecting the accuracy of symptom measurement.

In conclusion, while no significant relationship was found between psychomotor symptoms as measured by the PHQ-A and typing behaviour, our findings highlight the potential of typing behaviour signalling other depressive symptoms, such as changes in appetite and anhedonia disturbances. This study also underscores the importance of exploring individual depressive symptoms. By focusing on specific aspects of depression, researchers can gain more precise insights into how these symptoms manifest in typing behaviour. This, in turn, may allow for more effective monitoring and personalised mental health interventions. Future studies should focus on longitudinal data collection to capture the dynamic nature of depressive symptoms over time and consider integrating keystroke dynamics with other physiological or behavioural markers to enhance the accuracy and predictive power of digital phenotyping in depression.

### Citation diversity statement

The authors have attested that they made efforts to be mindful of diversity in selecting the citations used in this article.

## Supplementary information

Supplementary Material

## Data Availability

The data collected and analysed in the current study is not currently available to researchers outside of the approved team due to constraints placed on the project by the various ethics bodies. Additional related project documents are currently available from the web-based Australian and New Zealand Clinical Trials Registry.
