# Supplementary material for: Tapping into truth: an exploratory cross-sectional analysis of psychomotor symptoms and typing behaviour in an adolescent observational cohort
Source: NPP Digit Psychiatry Neurosci. 2025 Jun 18;3:14. doi: 10.1038/s44277-025-00033-0 (PMC12173933; doi:10.1038/s44277-025-00033-0)
Supplement: Supplementary file 1 — Supplementary Material [file 44277_2025_33_MOESM1_ESM.docx]

**Supplementary Material**

**Figure 1. Screenshot of text typing task**


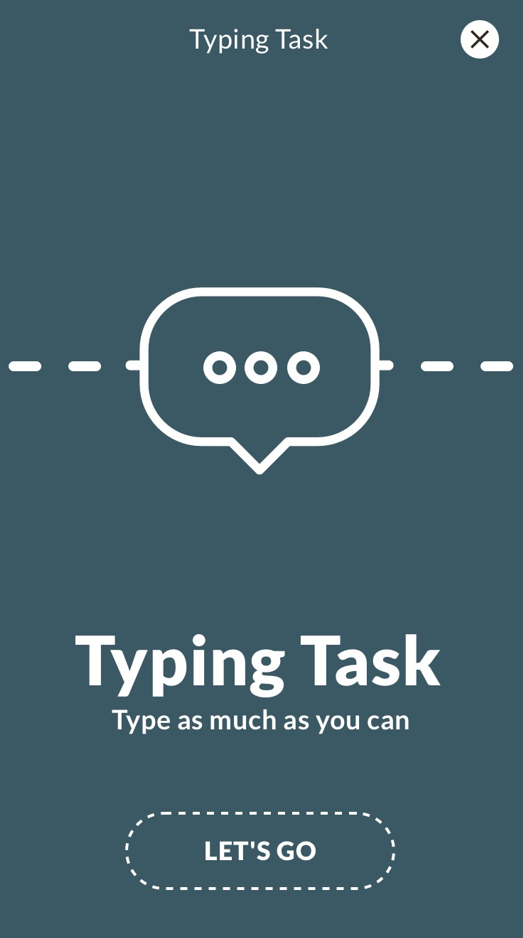

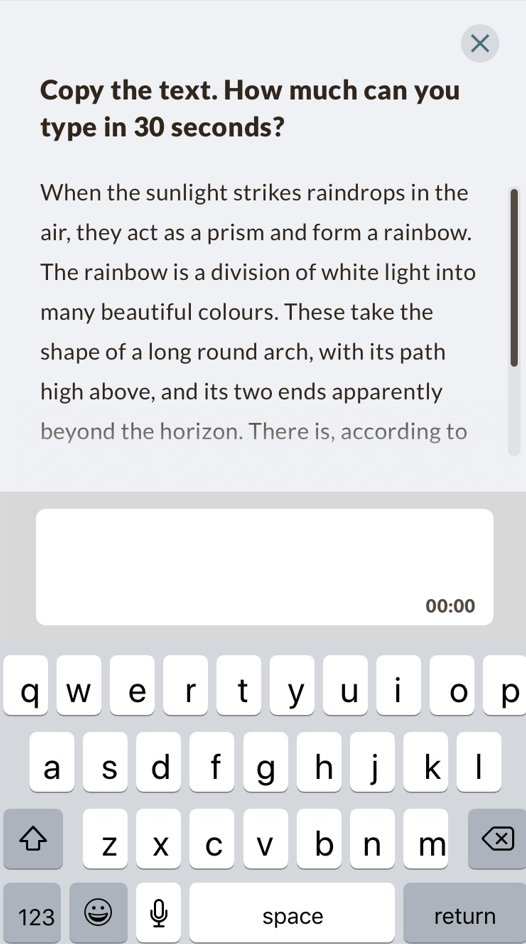


**Typing task scripts**

“For a minute or two she stood looking at the house, and wondering what to do next, when suddenly a footman in livery came running out of the wood and rapped loudly at the door with his knuckles. She considered him to be a footman because he was in livery. Otherwise, judging by his face only, she would have called him a fish. It was opened by another footman in livery, with a round face, and large eyes like a frog; and both footmen, Alice noticed, had powdered hair that curled all over their heads. She felt very curious to know what it was all about and crept a little way out of the wood to listen. The Fish-Footman began by producing from under his arm a great letter, nearly as large as himself, and this he handed over to the other.”

“There was a table set out under a tree in front of the house, and the March Hare and the Hatter were having tea at it. A Dormouse was sitting between them, fast asleep, and the other two were using it as a cushion, resting their elbows on it, and talking over its head. The table was a large one, but the three were all crowded together at one corner of it. Alice sat down in a large armchair at one end of the table. Alice looked all round the table, but there was nothing on it but tea. The party sat silent for a minute, while Alice thought over all she could remember about ravens and writing-desks, which wasn\'t much. The Hatter was the first to break the silence. He had taken his watch out of his pocket, and was looking at it uneasily, shaking it every now and then, and holding it to his ear.”

“So much I'll tell you, and no more. I were in Flint\'s ship when he buried the treasure, he and six along. Six strong seamen. They was ashore nigh on a week, and us standing off and on in the old Walrus. One fine day up went the signal, and here come Flint by himself in a little boat, and his head done up in a blue scarf. The sun was getting up, and mortal white he looked about the cutwater. But, there he was, you mind, and the six all dead. Dead and buried. How he done it, not a man aboard us could make out. It was battle, murder, and sudden death, leastways. Him against six. Billy Bones was the mate, Long John, he was quartermaster, and they asked him where the treasure was.”

“Then the sound of shouting arose again and the crackle of dried thorns. The enemy was breaking down the hedge. All the villagers swarmed to the point whence the crackling and the shouting came. They hurled stones over the hedges, and short arrows with flint heads. The children had never before seen men with the fighting light in their eyes. It was very strange and terrible and gave you a thick feeling in your throat. It was quite different from the pictures of fights in the illustrated papers at home. It seemed that the shower of stones had driven back the besiegers. The besieged drew breath, but at that moment the shouting and the crackling arose on the opposite side of the village and the crowd hastened to defend that point, and so the fight swayed to and fro across the village, for the besieged had not the sense to divide their forces as their enemies had done.”

“They were friends in a friendship closer than brotherhood. Nello was a little Ardennois. Patrasche was a big Fleming. They were both of the same age by length of years, yet one was still young, and the other was already old. They had dwelt together almost all their days. Both were orphaned and destitute, and owed their lives to the same hand. It had been the beginning of the tie between them, their first bond of sympathy; and it had strengthened day by day, and had grown with their growth, firm and indissoluble, until they loved one another very greatly. Their home was a little hut on the edge of a little village—a Flemish village a league from Antwerp, set amidst flat breadths of pasture and corn-lands, with long lines of poplars and of alders bending in the breeze on the edge of the great canal which ran through it.”

“The winter was very sharp already. That night, after they reached the hut, snow fell, and fell for very many days after that, so that the paths and the divisions in the fields were all obliterated, and all the smaller streams were frozen over, and the cold was intense upon the plains. Then, indeed, it became hard work to go round for the milk while the world was all dark, and carry it through the darkness to the silent town. Hard work, especially for Patrasche, for the passage of the years, that were only bringing Nello a stronger youth, were bringing him old age, and his joints were stiff and his bones ached often. But he would never give up his share of the labour. Nello would fain have spared him and drawn the cart himself, but Patrasche would not allow it.”

“A little girl, radiant and beautiful, shapely as a fairy and exquisitely dressed, was dancing gracefully in the middle of the lonely road, whirling slowly this way and that, her dainty feet twinkling in sprightly fashion. She was clad in flowing, fluffy robes of soft material that reminded Dorothy of woven cobwebs, only it was coloured in soft tintings of violet, rose, topaz, olive, azure, and white, mingled together most harmoniously in stripes which melted one into the other with soft blendings. Her hair was like spun gold and flowed around her in a cloud, no strand being fastened or confined by either pin or ornament or ribbon. Filled with wonder and admiration our friends approached and stood watching this fascinating dance. The girl was no taller than Dorothy, although more slender, nor did she seem any older than our little heroine.”

“But they by no means suddenly stopped. Little Walter Ashe\'s case proved to be rather a severe one, and after he had begun to mend, he caught cold somehow and was taken worse again. There were some serious symptoms, and for a few days Dr. Carr did not feel sure how things would turn. He did not speak of his anxiety at home, but kept silence and a cheerful face, as doctors know how to do. Only Katy, who was more intimate with her father than the rest, guessed that things were going gravely at the other house, and she was too well trained to ask questions. The threatening symptoms passed off, however, and little Walter slowly got better. But it was a long convalescence, and Mrs. Ashe grew thin and pale before he began to look rosy.”

**
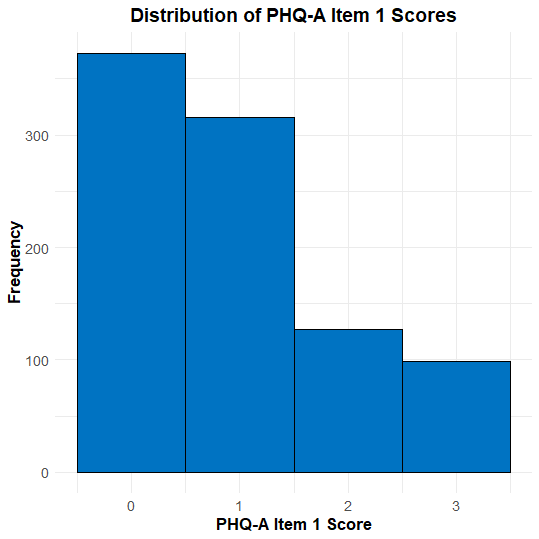
Figure 2. Distributions of PHQ-A items**


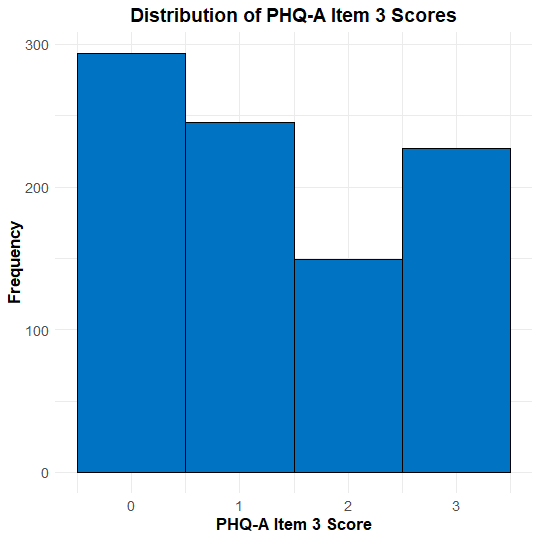

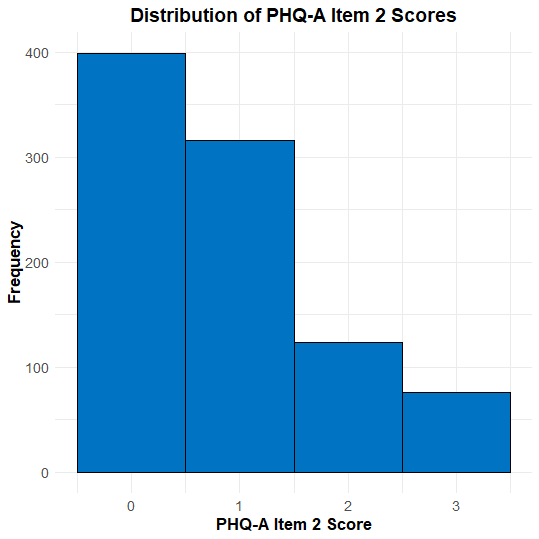

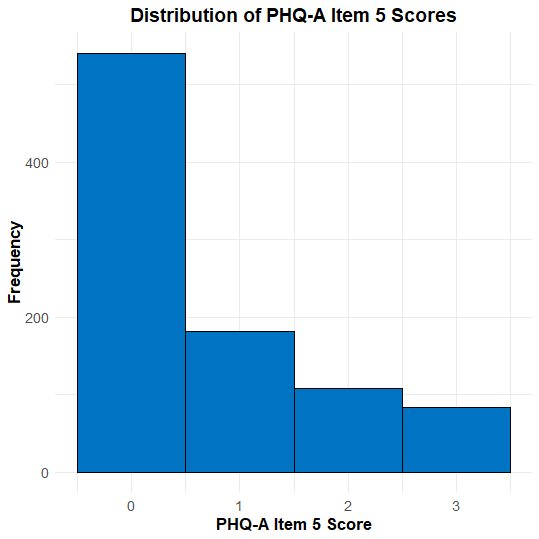
**
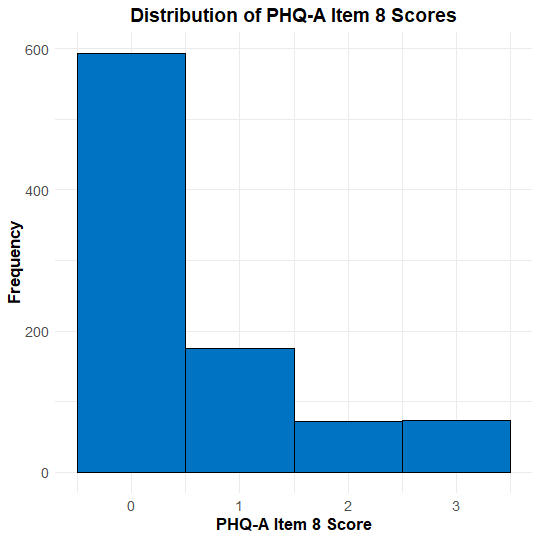
**
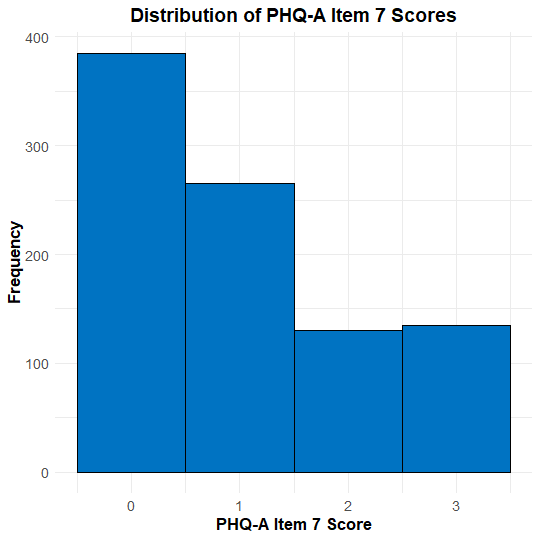


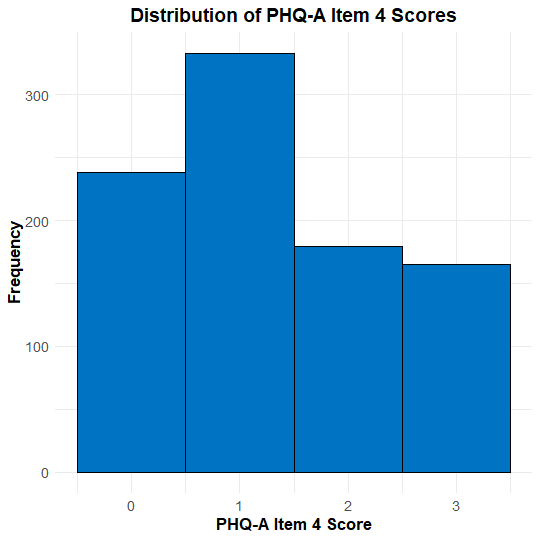


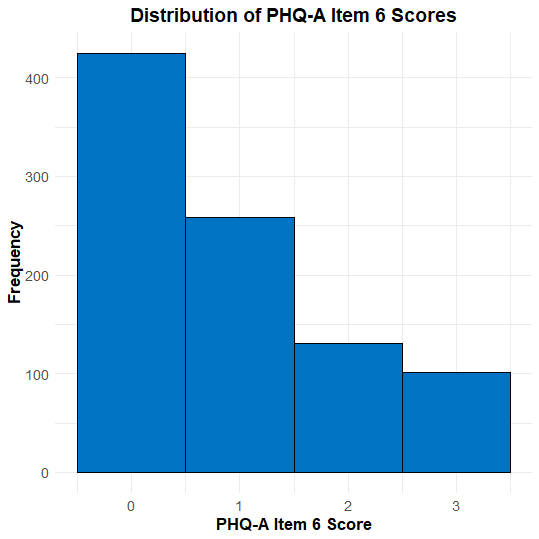


**
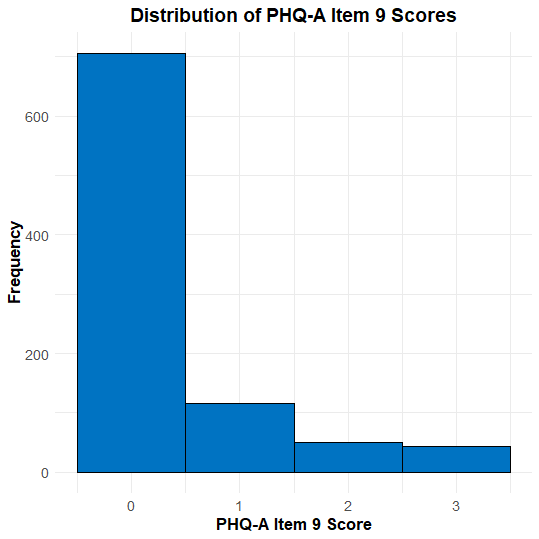
**

| ***PHQ-A Items*** | ***M (SD)*** |
| --- | --- |
| Anhedonia (PHQ-A item 1) | 0.96 (0.99) |
| Depressed mood (PHQ-A item 2) | 0.87 (0.94) |
| Sleep disturbance (PHQ-A item 3) | 1.35 (1.17) |
| Fatigue (PHQ-A item 4) | 1.30 (1.05) |
| Appetite/Weight changes (PHQ-A item 5) | 0.72 (1.00) |
| Feelings of worthlessness/guilt (PHQ-A item 6) | 0.91 (1.02) |
| Concentration difficulties (PHQ-A item 7) | 1.02 (1.07) |
| Psychomotor symptoms (PHQ-A item 8) | 0.59 (0.94) |
| Suicidal ideation (PHQ-A item 9) | 0.38 (0.80) |

**Table 1. Means and standard deviations of PHQ-A items**

**Table 2. Correlations between PHQ-A Items**

|  |  | **PHQ-A Item 1** | **PHQ-A Item 2** | **PHQ-A Item 3** | **PHQ-A Item 4** | **PHQ-A Item 5** | **PHQ-A Item 6** | **PHQ-A Item 7** | **PHQ-A Item 8** | **PHQ-A Item 9** |
| --- | --- | --- | --- | --- | --- | --- | --- | --- | --- | --- |
| Anhedonia (PHQ-A item 1) | ***r*** | 1 |  |  |  |  |  |  |  |  |
|  | ***p*** | - |  |  |  |  |  |  |  |  |
| Depressed mood (PHQ-A item 2) | ***r*** | 0.45 | 1 |  |  |  |  |  |  |  |
|  | ***p*** | <.001 | - |  |  |  |  |  |  |  |
| Sleep disturbance (PHQ-A item 3) | ***r*** | 0.38 | 0.51 | 1 |  |  |  |  |  |  |
|  | ***p*** | <.001 | <.001 | - |  |  |  |  |  |  |
| Fatigue (PHQ-A item 4) | ***r*** | 0.39 | 0.53 | 0.59 | 1 |  |  |  |  |  |
|  | ***p*** | <.001 | <.001 | <.001 | - |  |  |  |  |  |
| Appetite/Weight changes (PHQ-A item 5) | ***r*** | 0.36 | 0.51 | 0.47 | 0.45 | 1 |  |  |  |  |
|  | ***p*** | <.001 | <.001 | <.001 | <.001 | **-** |  |  |  |  |
| Feelings of worthlessness/guilt (PHQ-A item 6) | ***r*** | 0.39 | 0.69 | 0.48 | 0.49 | 0.54 | 1 |  |  |  |
|  | ***p*** | <.001 | <.001 | <.001 | <.001 | <.001 | **-** |  |  |  |
| Concentration difficulties (PHQ-A item 7) | ***r*** | 0.40 | 0.54 | 0.55 | 0.50 | 0.47 | 0.48 | 1 |  |  |
|  | ***p*** | <.001 | <.001 | <.001 | <.001 | <.001 | <.001 | **-** |  |  |
| Psychomotor symptoms (PHQ-A item 8) | ***r*** | 0.34 | 0.54 | 0.45 | 0.46 | 0.47 | 0.49 | 0.54 | 1 |  |
|  | ***p*** | <.001 | <.001 | <.001 | <.001 | <.001 | <.001 | <.001 | **-** |  |
| Suicidal ideation (PHQ-A item 9) | ***r*** | 0.31 | 0.64 | 0.38 | 0.39 | 0.46 | 0.60 | 0.39 | 0.49 | 1 |
|  | ***p*** | <.001 | <.001 | <.001 | <.001 | <.001 | <.001 | <.001 | <.001 | **-** |

**Table 3. Features, calculation formulas, and descriptions of keystroke features.**

| **Features** | **Calculation Formula** | **Description** |
| --- | --- | --- |
| **Keystroke Timing Features** |  |  |
| Dwell | Median(Key Release Time – Key Press Time) | Median time a key is held down |
| Latency | Median(Key Release Time (n+1) – Key Press Time (n)) | Median time from initiating one keystroke to finishing the next |
| Interval | Median(Key Press Time (n+1) – Key Release Time (n)) | Median time between releasing one key and pressing the next |
| Down-down time | Median(Key Press Time (n+1) – Key Press Time (n)) | Median time between consecutive key presses |
| Up-up time | Median(Key Release Time (n+1) – Key Release Time (n)) | Median time between successive key releases |
| **Keystroke Frequency Features** |  |  |
| Total Keystrokes | Count(All keystrokes) | Overall number of keys pressed |
| Total Spaces | Count(Space keys) | Frequency of spaces inserted between words |
| Total Backspaces | Count(Backspace keys) | Frequency of error correction via backspaces |
| Proportion of Spaces | Total Backspaces / Total Keystrokes | Proportion of key presses that are backspaces |
| Proportion of Backspaces | Total Spaces / Total Keystrokes | Proportion of key presses that are spaces |

*Note:* All timing features are measured in milliseconds (ms).

**Table 4. Correlations between keystroke timing features**

|  |  | **Median Dwell** | **Median Interval** | **Median Latency** | **Median Down-down time** | **Median Up-up time** |
| --- | --- | --- | --- | --- | --- | --- |
| **Median Dwell** | ***r*** | 1 |  |  |  |  |
|  | ***p*** | - |  |  |  |  |
| **Median Latency** | ***r*** | .53 | 1 |  |  |  |
|  | ***p*** | <.001 | - |  |  |  |
| **Median Interval** | ***r*** | .20 | .92 | 1 |  |  |
|  | ***p*** | <.001 | <.001 | - |  |  |
| **Median Down-down time** | ***r*** | 0.38 | 0.97 | 0.97 | 1 |  |
|  | ***p*** | <.001 | <.001 | <.001 | - |  |
| **Median Up-up time** | ***r*** | 0.39 | 0.98 | 0.97 | 0.98 | 1 |
|  | ***p*** | <.001 | <.001 | <.001 | <.001 | **-** |

**Table 5. Correlations between keystroke frequency features**

|  |  | **Total Keystrokes** | **Total Spaces** | **Spaces Ratio** | **Total Backspaces** | **Backspaces Ratio** |
| --- | --- | --- | --- | --- | --- | --- |
| **Total Keystrokes** | ***r*** | 1 |  |  |  |  |
|  | ***p*** | - |  |  |  |  |
| **Total Spaces** | ***r*** | .89 | 1 |  |  |  |
|  | ***p*** | <.001 | - |  |  |  |
| **Spaces Ratio** | ***r*** | .13 | .53 | 1 |  |  |
|  | ***p*** | <.001 | <.001 | - |  |  |
| **Total Backspaces** | ***r*** | .28 | .04 | -.39 | 1 |  |
|  | ***p*** | <.001 | .208 | <.001 | - |  |
| **Backspaces Ratio** | ***r*** | -.14 | -.31 | -.47 | .84 | 1 |
|  | ***p*** | <.001 | <.001 | <.001 | <.001 | - |

**Figure 3. Distributions of skewed data before and after transformation**

Due to no zero values being included in the median down-down time distribution, data were transformed using the natural log of median down-down time values.


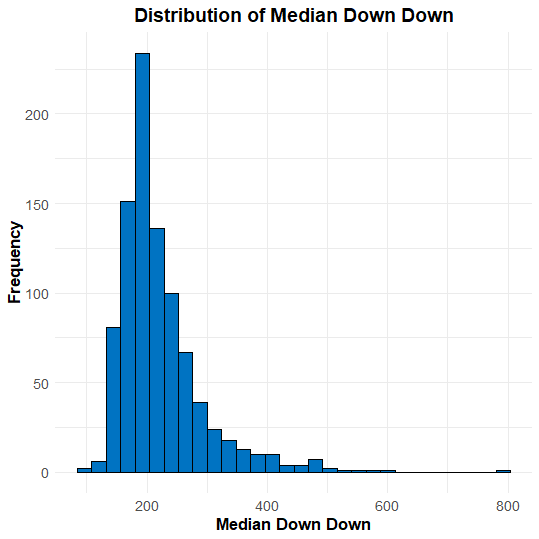
 **
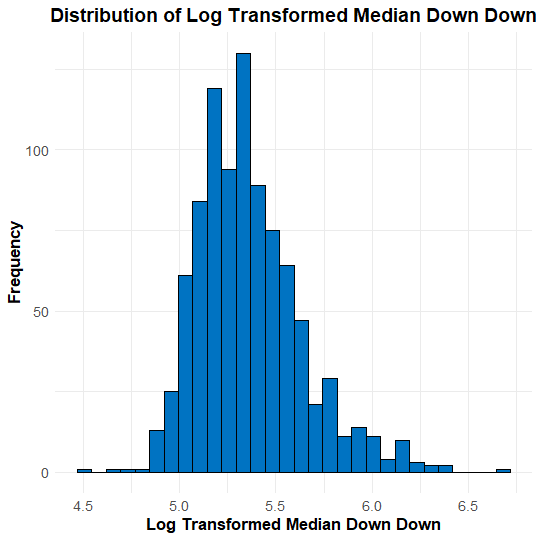
**

Due to zero values being included in the distributions, total backspace and backspace ratio values were transformed using the square root of their values.

**
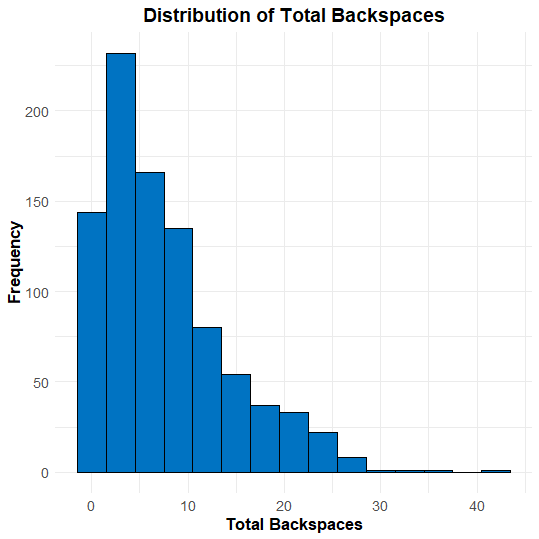
** **
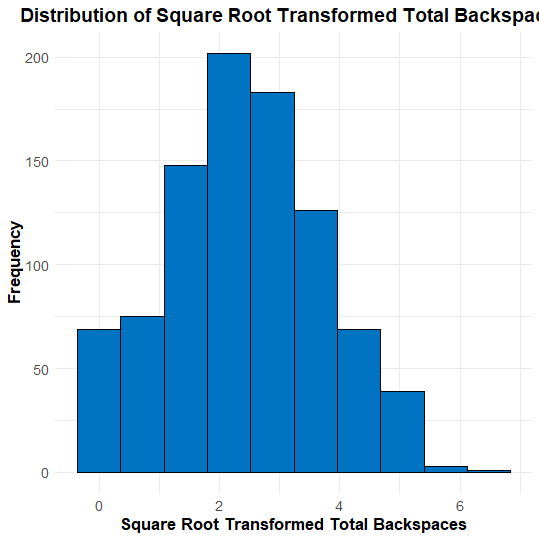
**

**
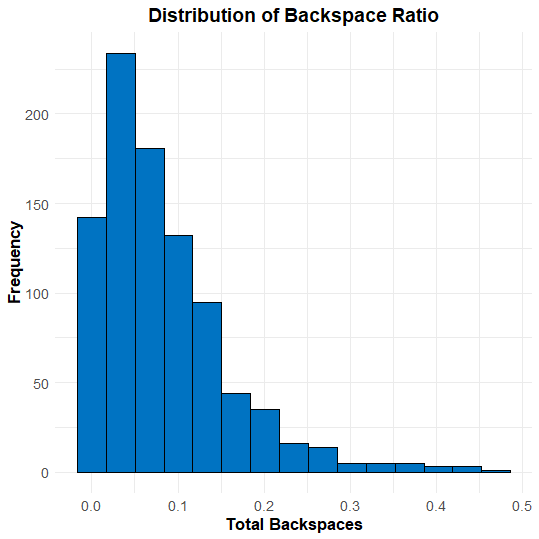
**
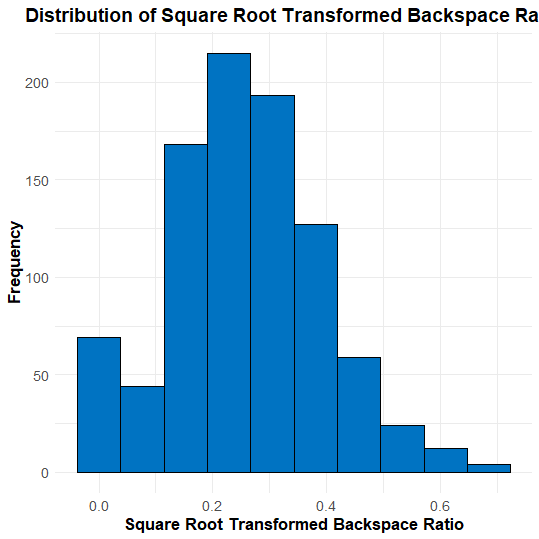


|  | *Main Analysis* | | | | | | *Sensitivity Analysis* | | |
| --- | --- | --- | --- | --- | --- | --- | --- | --- | --- |
| Variable | Coefficient  (*b*) | Standard Error (SE) | 95% CI (Lower) | 95% CI (Upper) | *t* | *p* | Coefficient  (*b*) | *t* | *p* |
| Intercept \| Prose category (random effect)* | - | 0.07 | - | - | - | - |  |  |  |
| Intercept (fixed effect) | 5.69 | 0.24 | 5.11 | 6.26 | 23.49 | <.001 | 5.69 | 27.93 | <.001 |
| PHQA - Item 1 | 0.01 | 0.01 | -0.01 | 0.04 | 1.21 | 0.265 | 0.02 | 2.18 | 0.030 |
| PHQA - Item 2 | 0.01 | 0.01 | -0.02 | 0.03 | 0.58 | 0.582 | 0.0007 | 0.05 | 0.961 |
| PHQA - Item 3 | 0.004 | 0.01 | -0.01 | 0.02 | 0.52 | 0.619 | -0.001 | -0.14 | 0.888 |
| PHQA - Item 4 | -0.01 | 0.01 | -0.03 | 0.01 | -0.91 | 0.394 | -0.01 | -0.57 | 0.566 |
| **PHQA - Item 5** | -0.02 | 0.01 | -0.05 | 0.00 | -2.41 | 0.048 | -0.03 | -2.47 | 0.014 |
| PHQA - Item 6 | -0.004 | 0.02 | -0.04 | 0.03 | -0.27 | 0.797 | -0.003 | -0.27 | 0.788 |
| PHQA - Item 7 | 0.004 | 0.02 | -0.03 | 0.04 | 0.24 | 0.816 | 0.003 | 0.32 | 0.751 |
| PHQA - Item 8 | -0.01 | 0.01 | -0.04 | 0.02 | -0.99 | 0.358 | -0.01 | -0.77 | 0.439 |
| PHQA - Item 9 | 0.01 | 0.01 | -0.01 | 0.04 | 1.65 | 0.146 | 0.01 | 0.67 | 0.503 |
| Handedness (Left hand) | 0.07 | 0.10 | -0.26 | 0.40 | 0.68 | 0.546 | 0.06 | 0.61 | 0.539 |
| Handedness (Not reported) | -0.001 | 0.02 | -0.05 | 0.05 | -0.06 | 0.950 | -0.003 | -0.18 | 0.860 |
| Handedness (Right hand) | 0.16 | 0.04 | 0.05 | 0.26 | 3.53 | 0.010 | 0.13 | 3.94 | <.001 |
| Sex (Male) | 0.11 | 0.02 | 0.08 | 0.15 | 7.50 | <.001 | 0.11 | 6.00 | <.001 |
| Age | -0.02 | 0.02 | -0.07 | 0.02 | -1.22 | 0.264 | -0.02 | -1.64 | 0.102 |
| Location (Major Cities) | -0.08 | 0.03 | -0.14 | -0.02 | -2.97 | 0.021 | -0.08 | -4.09 | <.001 |
| Location (Outer Regional) | 0.02 | 0.04 | -0.07 | 0.11 | 0.52 | 0.621 | 0.01 | 0.31 | 0.759 |
| English spoken at home (True) | -0.0004 | 0.04 | -0.09 | 0.09 | -0.01 | 0.991 | 0.002 | 0.06 | 0.956 |
| Days between assessment and typing task | 0.004 | 0.002 | -0.0002 | 0.007 | 2.23 | 0.063 | 0.003 | 1.17 | 0.243 |

**Table 6. PHQ-A individual items and down-down time model**

Note: *****SD is reported for random effect (not SE)

**Figure 4. Diagnostic Plots for PHQ-A individual items and down-down time model**


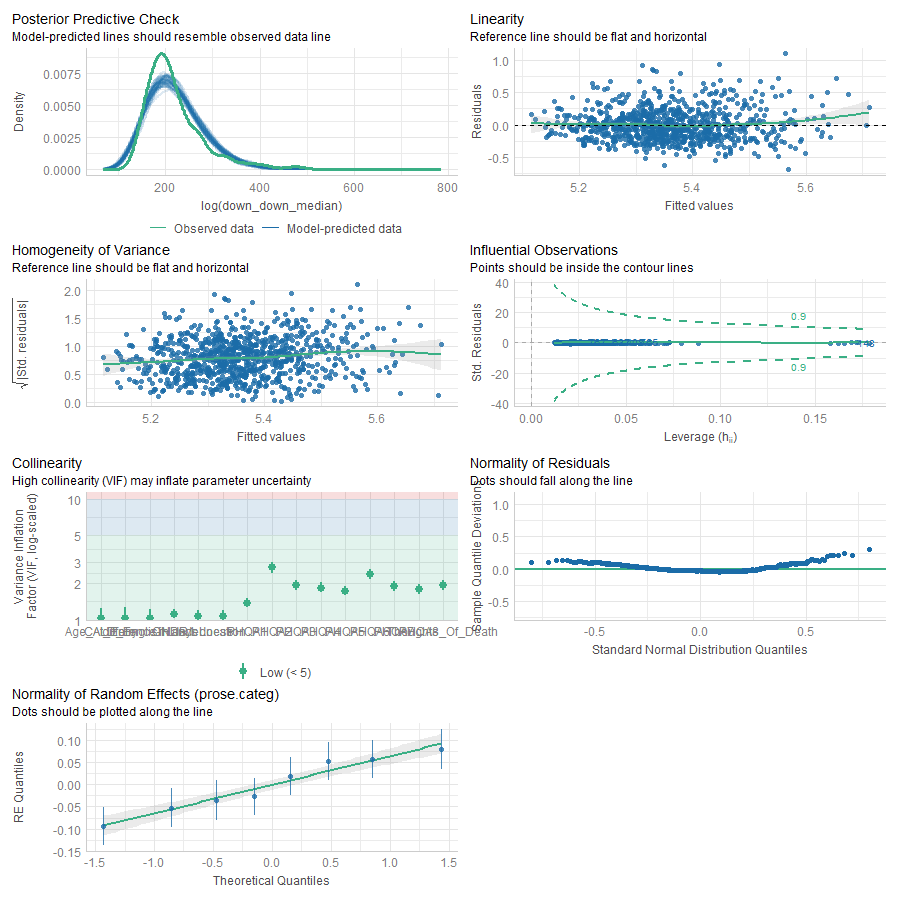

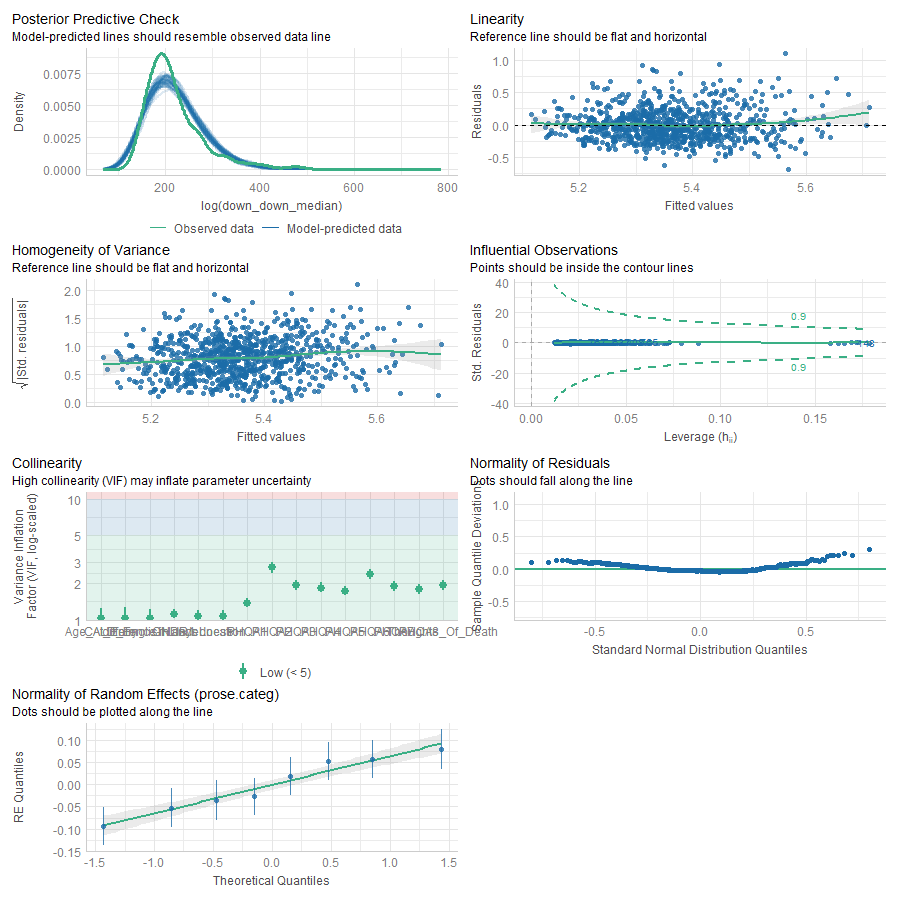


**
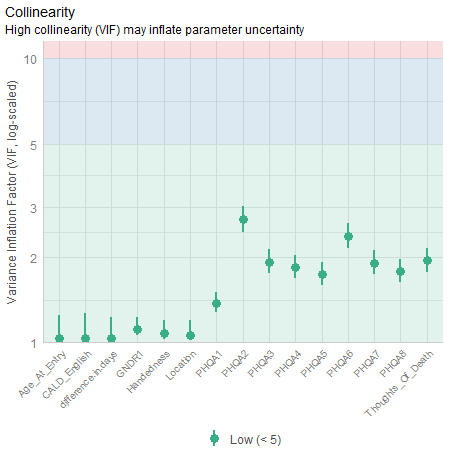
**

**Table 7. SDE total score and down-down time model**

|  | *Main Analysis* | | | | | | *Sensitivity Analysis* | | |
| --- | --- | --- | --- | --- | --- | --- | --- | --- | --- |
| Variable | Coefficient  (*b*) | Standard Error (SE) | 95% CI (Lower) | 95% CI (Upper) | *t* | *p* | Coefficient  (*b*) | *t* | *p* |
| Intercept \| Prose category (random effect)* | - | 0.07 | - | - | - | - | - | - | - |
| Intercept (fixed effect) | 5.73 | 0.19 | 5.28 | 6.18 | 30.16 | <.001 | 5.73 | 28.09 | <.001 |
| **SDE total score** | -0.02 | 0.01 | -0.03 | 0.00 | -2.39 | 0.049 | -0.02 | -2.86 | 0.004 |
| Handedness (Left hand) | 0.08 | 0.11 | -0.25 | 0.41 | 0.75 | 0.508 | 0.06 | 0.64 | 0.525 |
| Handedness (Not reported) | -0.001 | 0.02 | -0.05 | 0.04 | -0.07 | 0.949 | -0.003 | -0.16 | 0.874 |
| Handedness (Right hand) | 0.16 | 0.05 | 0.04 | 0.28 | 3.16 | 0.017 | 0.13 | 3.77 | <.001 |
| Sex (Male) | 0.10 | 0.01 | 0.07 | 0.14 | 7.67 | <.001 | 0.1 | 5.22 | <.001 |
| Age | -0.02 | 0.02 | -0.06 | 0.01 | -1.49 | 0.180 | -0.02 | -1.69 | 0.091 |
| Location (Major Cities) | -0.08 | 0.02 | -0.13 | -0.02 | -3.33 | 0.013 | -0.08 | -4.12 | <.001 |
| Location (Outer Regional) | 0.02 | 0.04 | -0.08 | 0.12 | 0.47 | 0.655 | 0.02 | 0.34 | 0.732 |
| English spoken at home (True) | -0.01 | 0.04 | -0.09 | 0.07 | -0.28 | 0.788 | -0.01 | -0.21 | 0.834 |
| Days between assessment and typing task | 0.005 | 0.002 | 0.0001 | 0.01 | 2.45 | 0.045 | 0.004 | 1.44 | 0.149 |

Note: *****SD is reported for random effect (not SE)

**Figure 5. Diagnostic Plots for SDE total score and down-down time model**


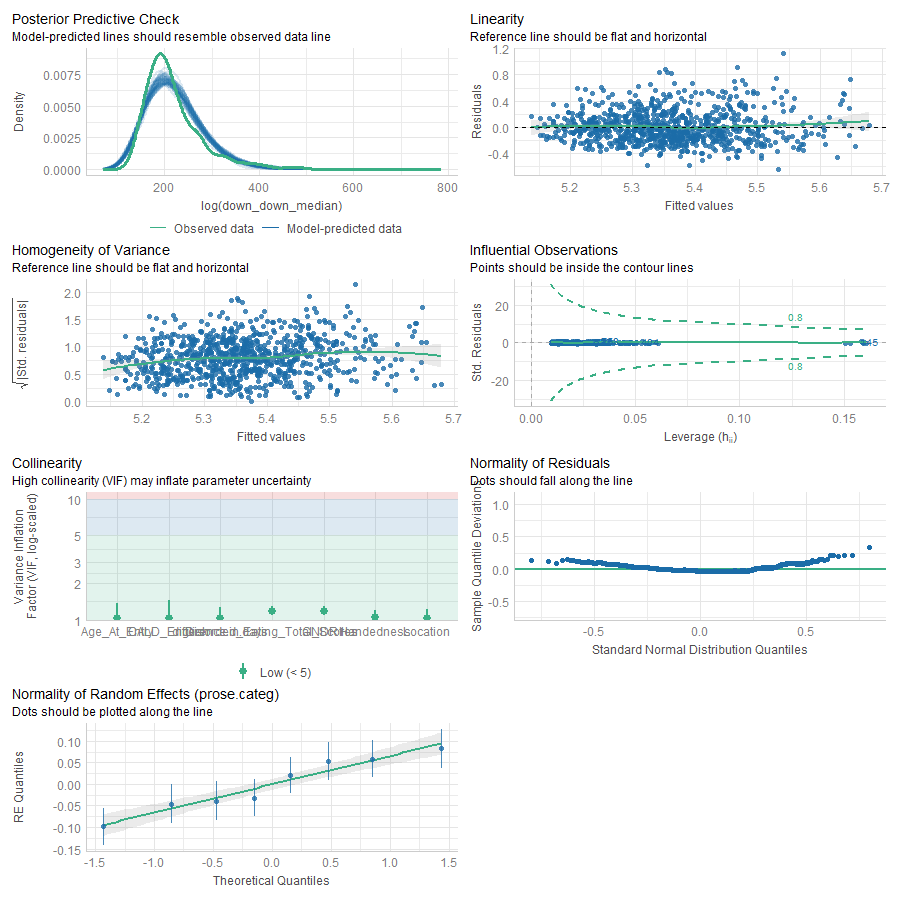

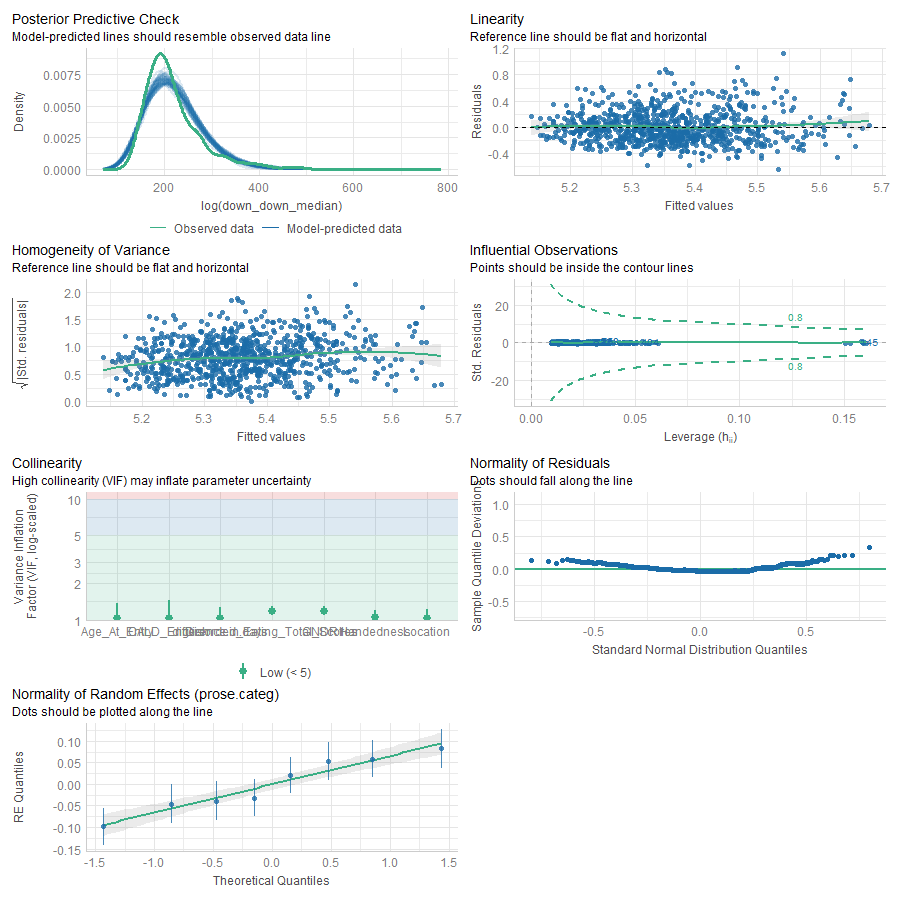


**
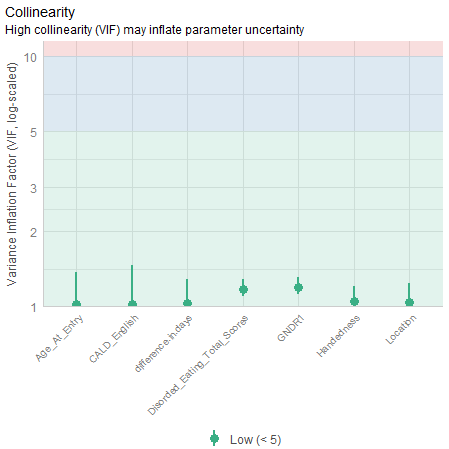
**

**Table 8. SDE positive screening and down-down time model**

|  | *Main Analysis* | | | | | | *Sensitivity Analysis* | | |
| --- | --- | --- | --- | --- | --- | --- | --- | --- | --- |
| Variable | Coefficient  (*b*) | Standard Error (SE) | 95% CI (Lower) | 95% CI (Upper) | *t* | *p* | Coefficient  (*b*) | *t* | *p* |
| Intercept \| Prose category (random effect)* | - | 0.07 | - | - | - | - | - | - | - |
| Intercept (fixed effect) | 5.73 | 0.20 | 5.25 | 6.20 | 28.62 | <.001 | 5.74 | 28.1 | <.001 |
| **SDE positive screening** | -0.05 | 0.01 | -0.08 | -0.02 | -3.76 | 0.007 | -0.05 | -3.00 | 0.003 |
| Handedness (Left hand) | 0.07 | 0.10 | -0.26 | 0.39 | 0.65 | 0.558 | 0.05 | 0.52 | 0.606 |
| Handedness (Not reported) | -0.002 | 0.02 | -0.05 | 0.04 | -0.11 | 0.916 | -0.004 | -0.20 | 0.844 |
| Handedness (Right hand) | 0.16 | 0.05 | 0.04 | 0.28 | 3.14 | 0.018 | 0.13 | 3.75 | <.001 |
| Sex (Male) | 0.11 | 0.01 | 0.07 | 0.14 | 7.92 | <.001 | 0.10 | 5.37 | <.001 |
| Age | -0.02 | 0.02 | -0.06 | 0.01 | -1.48 | 0.183 | -0.03 | -1.74 | 0.083 |
| Location (Major Cities) | -0.08 | 0.02 | -0.14 | -0.02 | -3.39 | 0.012 | -0.08 | -4.22 | <.001 |
| Location (Outer Regional) | 0.02 | 0.04 | -0.08 | 0.12 | 0.41 | 0.700 | 0.01 | 0.28 | 0.781 |
| English spoken at home (True) | -0.01 | 0.04 | -0.09 | 0.08 | -0.27 | 0.798 | -0.01 | -0.19 | 0.846 |
| Days between assessment and typing task | 0.005 | 0.002 | 0.0001 | 0.01 | 2.43 | 0.046 | 0.004 | 1.48 | 0.139 |

Note: *****SD is reported for random effect (not SE)

**Figure 6. Diagnostic Plots for SDE positive screening and down-down time model**


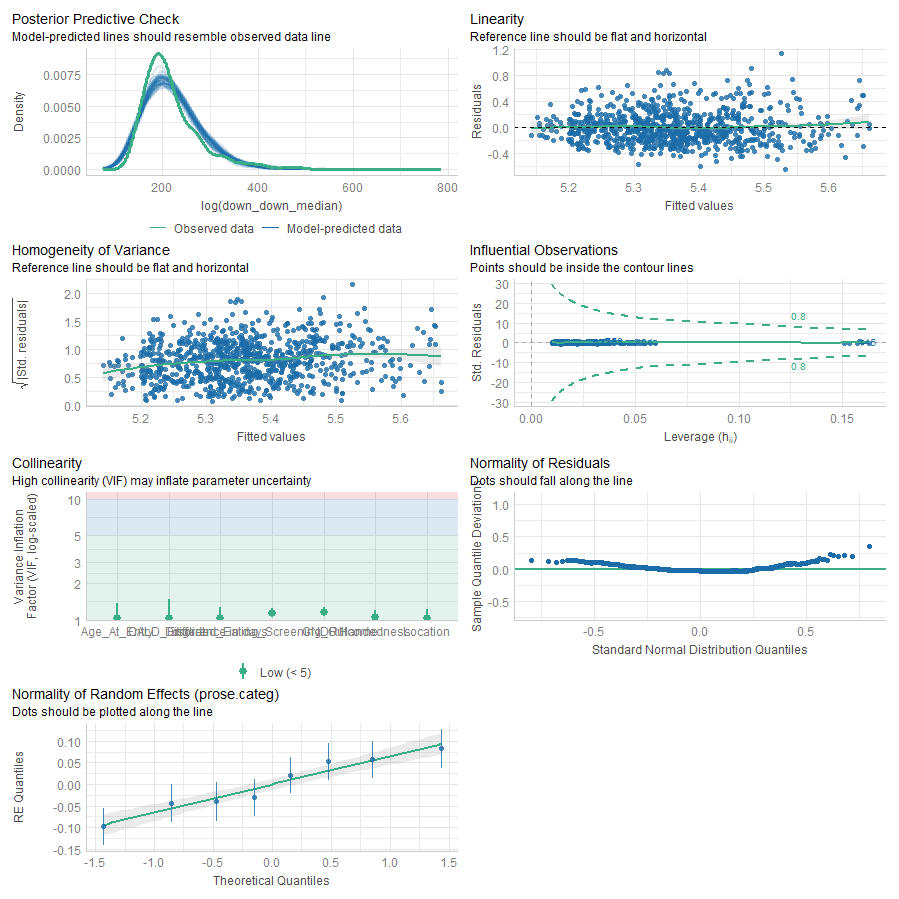

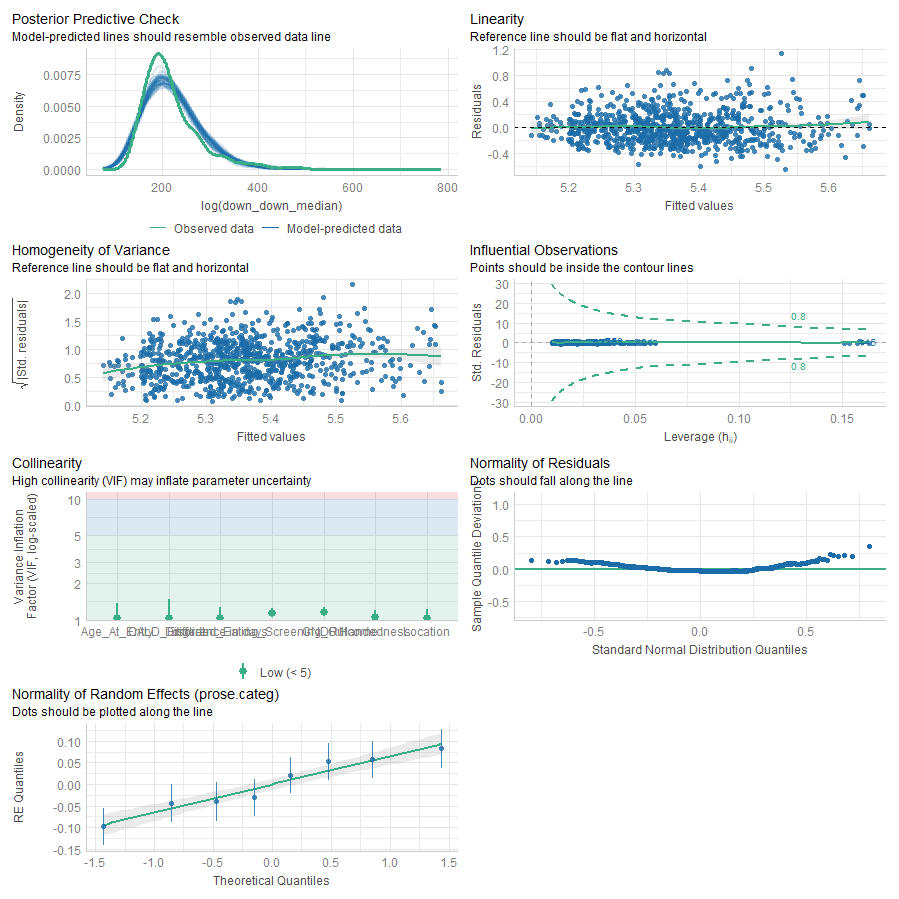


**
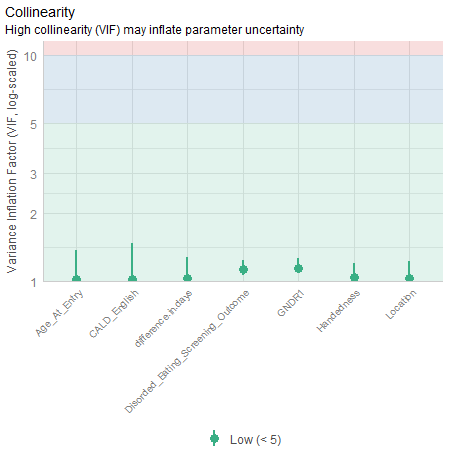
**

|  | *Main Analysis* | | | | | | *Sensitivity Analysis* | | |
| --- | --- | --- | --- | --- | --- | --- | --- | --- | --- |
| Variable | Coefficient  (*b*) | Standard Error (SE) | 95% CI (Lower) | 95% CI (Upper) | *t* | *p* | Coefficient  (*b*) | *t* | *p* |
| Intercept \| Prose category (random effect)* | - | 0.07 | - | - | - | - |  |  |  |
| Intercept (fixed effect) | 5.72 | 0.19 | 5.26 | 6.18 | 29.43 | <.001 | 5.72 | 28.10 | <.001 |
| SDE item 1 | 0.0002 | 0.01 | -0.03 | 0.03 | 0.02 | 0.988 | 0.004 | 0.23 | 0.818 |
| SDE item 2 | 0.01 | 0.02 | -0.04 | 0.06 | 0.43 | 0.680 | 0.004 | 0.20 | 0.840 |
| SDE item 3 | -0.05 | 0.04 | -0.14 | 0.03 | -1.48 | 0.184 | -0.04 | -1.04 | 0.298 |
| **SDE item 4** | -0.06 | 0.02 | -0.12 | 0.00 | -2.46 | 0.045 | -0.05 | -2.39 | 0.017 |
| SDE item 5 | -0.001 | 0.01 | -0.02 | 0.02 | -0.15 | 0.883 | -0.01 | -0.55 | 0.581 |
| Handedness (Left hand) | 0.09 | 0.10 | -0.23 | 0.40 | 0.84 | 0.459 | 0.06 | 0.67 | 0.506 |
| Handedness (Not reported) | 0.001 | 0.02 | -0.05 | 0.05 | 0.07 | 0.949 | -0.002 | -0.08 | 0.937 |
| Handedness (Right hand) | 0.16 | 0.05 | 0.04 | 0.28 | 3.24 | 0.015 | 0.13 | 3.71 | <.001 |
| Sex (Male) | 0.10 | 0.02 | 0.06 | 0.14 | 6.28 | <.001 | 0.09 | 4.88 | <.001 |
| Age | -0.02 | 0.02 | -0.06 | 0.02 | -1.42 | 0.199 | -0.02 | -1.63 | 0.104 |
| Location (Major Cities) | -0.08 | 0.02 | -0.13 | -0.02 | -3.17 | 0.016 | -0.08 | -4.00 | <.001 |
| Location (Outer Regional) | 0.02 | 0.04 | -0.09 | 0.12 | 0.42 | 0.690 | 0.01 | 0.31 | 0.760 |
| English spoken at home (True) | -0.01 | 0.04 | -0.10 | 0.07 | -0.41 | 0.695 | -0.01 | -0.34 | 0.736 |
| Days between assessment and typing task | 0.005 | 0.002 | 0.0004 | 0.01 | 2.61 | 0.036 | 0.004 | 1.5 | 0.134 |

**Table 9. SDE individual items and down-down time model**

Note: *****SD is reported for random effect (not SE)

**Figure 7. Diagnostic Plots for SDE individual items and down-down time model**


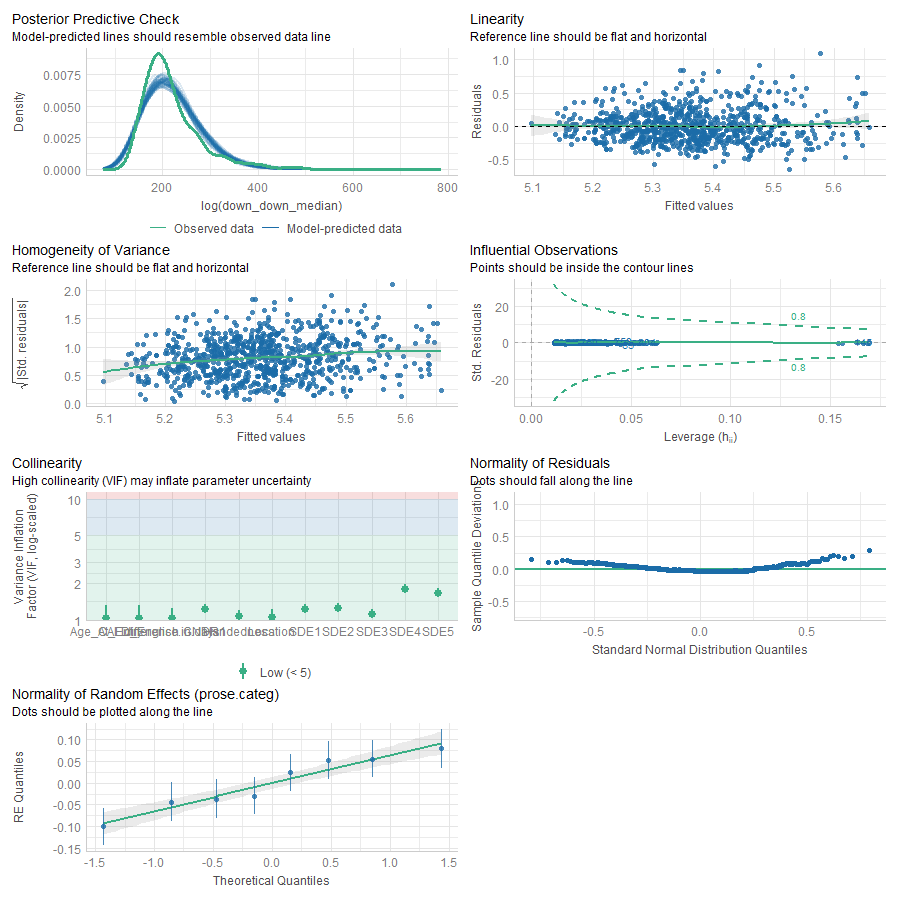

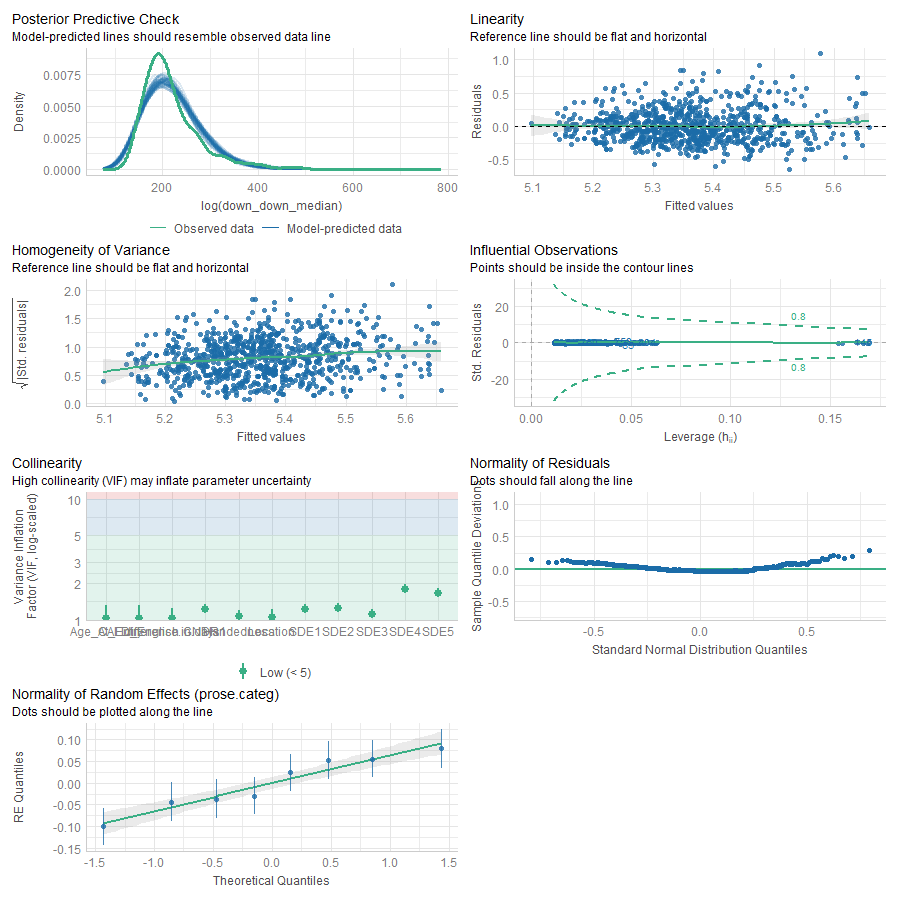


**
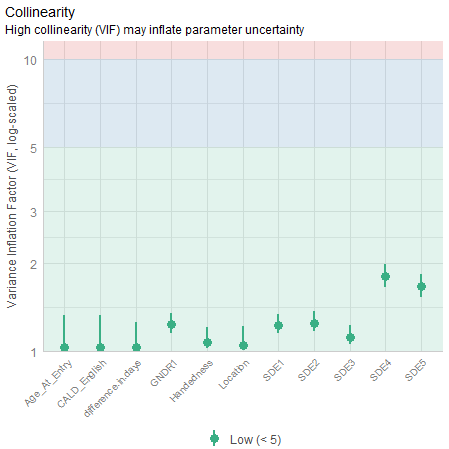
**

|  | *Main Analysis* | | | | | | *Sensitivity Analysis* | | |
| --- | --- | --- | --- | --- | --- | --- | --- | --- | --- |
| Variable | Coefficient  (*b*) | Standard Error (SE) | 95% CI (Lower) | 95% CI (Upper) | *t* | *p* | Coefficient  (*b*) | *t* | *p* |
| Intercept \| Prose category (random effect)* | - | 0.07 | - | - | - | - | - | - | - |
| Intercept (fixed effect) | 5.71 | 0.23 | 5.17 | 6.26 | 24.81 | <.001 | 5.72 | 27.99 | <.001 |
| ISI total score | -0.002 | 0.001 | -0.004 | 0.001 | -1.24 | 0.256 | -0.002 | -1.61 | 0.109 |
| Handedness (Left hand) | 0.07 | 0.11 | -0.26 | 0.40 | 0.69 | 0.538 | 0.06 | 0.63 | 0.531 |
| Handedness (Not reported) | -0.005 | 0.02 | -0.05 | 0.04 | -0.24 | 0.821 | -0.01 | -0.31 | 0.757 |
| Handedness (Right hand) | 0.15 | 0.05 | 0.04 | 0.27 | 3.17 | 0.017 | 0.12 | 3.57 | <.001 |
| Sex (Male) | 0.12 | 0.02 | 0.08 | 0.15 | 7.54 | <.001 | 0.11 | 6.09 | <.001 |
| Age | -0.02 | 0.02 | -0.07 | 0.02 | -1.38 | 0.212 | -0.03 | -1.75 | 0.080 |
| Location (Major Cities) | -0.08 | 0.03 | -0.14 | -0.02 | -2.98 | 0.021 | -0.08 | -4.11 | <.001 |
| Location (Outer Regional) | 0.01 | 0.04 | -0.09 | 0.12 | 0.31 | 0.765 | 0.01 | 0.23 | 0.819 |
| English spoken at home (True) | -0.01 | 0.04 | -0.09 | 0.08 | -0.15 | 0.887 | -0.001 | -0.03 | 0.974 |
| Days between assessment and typing task | 0.004 | 0.002 | 0.0001 | 0.01 | 2.38 | 0.050 | 0.003 | 1.16 | 0.245 |

**Table 10. ISI total score and down-down time model**

Note: *****SD is reported for random effect (not SE)

**Figure 8. Diagnostic Plots for ISI total score and down-down time model**


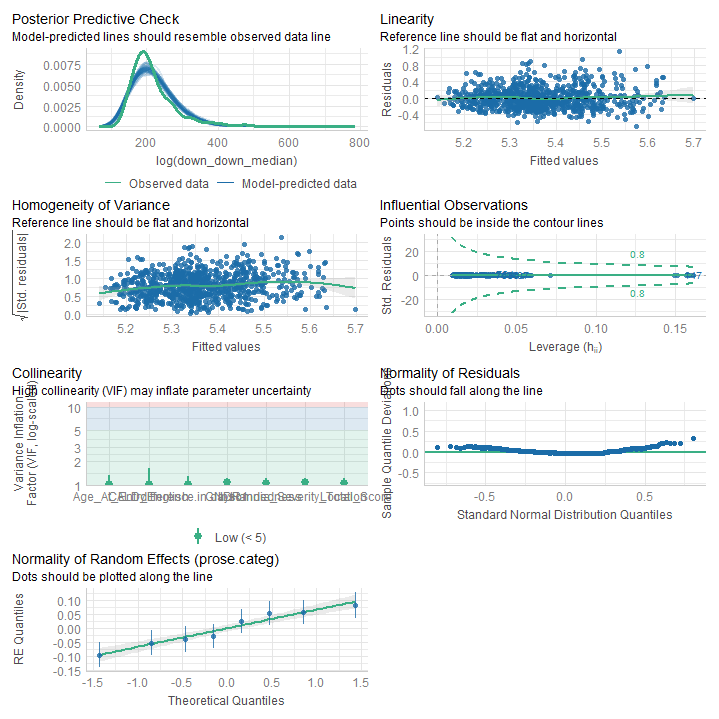

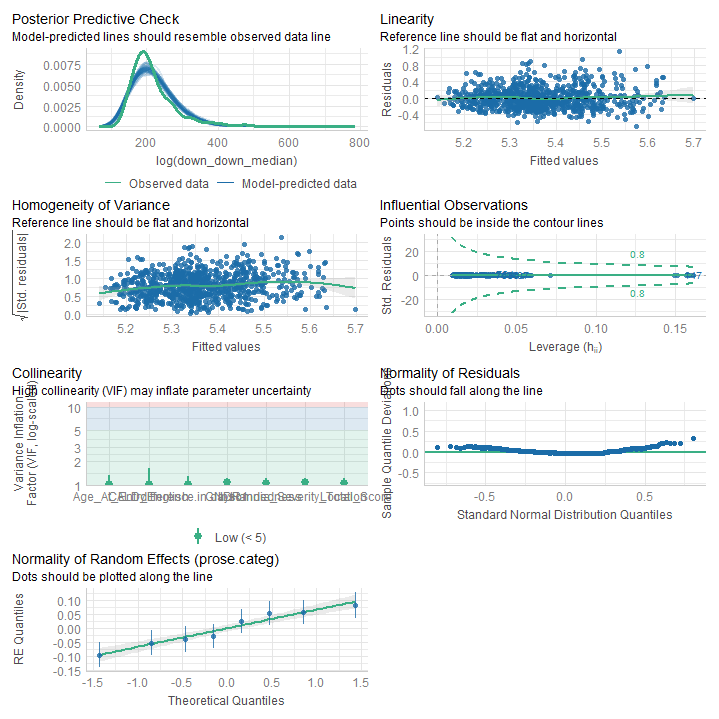


**
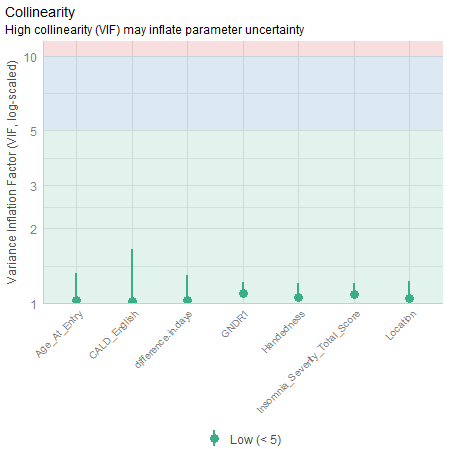
**

|  | *Main Analysis* | | | | | | *Sensitivity Analysis* | | |
| --- | --- | --- | --- | --- | --- | --- | --- | --- | --- |
| Variable | Coefficient  (*b*) | Standard Error (SE) | 95% CI (Lower) | 95% CI (Upper) | *t* | *p* | Coefficient  (*b*) | *t* | *p* |
| Intercept \| Prose category (random effect)* | - | 0.07 | - | - | - | - |  |  |  |
| Intercept (fixed effect) | 5.70 | 0.22 | 5.17 | 6.23 | 25.59 | <.001 | 5.71 | 27.93 | <.001 |
| CAS-8 total score | -0.0009 | 0.001 | -0.004 | 0.002 | -0.65 | 0.539 | -0.002 | -0.95 | 0.340 |
| Handedness (Left hand) | 0.07 | 0.10 | -0.26 | 0.40 | 0.66 | 0.557 | 0.05 | 0.55 | 0.584 |
| Handedness (Not reported) | -0.004 | 0.02 | -0.05 | 0.04 | -0.21 | 0.836 | -0.005 | -0.25 | 0.801 |
| Handedness (Right hand) | 0.15 | 0.05 | 0.04 | 0.27 | 3.12 | 0.018 | 0.12 | 3.54 | <.001 |
| Sex (Male) | 0.12 | 0.01 | 0.09 | 0.15 | 8.91 | <.001 | 0.11 | 5.92 | <.001 |
| Age | -0.02 | 0.02 | -0.07 | 0.02 | -1.35 | 0.218 | -0.02 | -1.70 | 0.090 |
| Location (Major Cities) | -0.08 | 0.03 | -0.14 | -0.02 | -2.98 | 0.021 | -0.08 | -4.10 | <.001 |
| Location (Outer Regional) | 0.01 | 0.04 | -0.09 | 0.12 | 0.34 | 0.744 | 0.01 | 0.27 | 0.787 |
| English spoken at home (True) | -0.01 | 0.04 | -0.09 | 0.08 | -0.14 | 0.895 | -0.008 | -0.02 | 0.981 |
| Days between assessment and typing task | 0.004 | 0.002 | 0.0001 | 0.01 | 2.42 | 0.047 | 0.003 | 1.17 | 0.242 |

**Table 11. CAS-8 total score and down-down time model**

Note: *****SD is reported for random effect (not SE)

**Figure 9. Diagnostic Plots for CAS-8 total score and down-down time model**


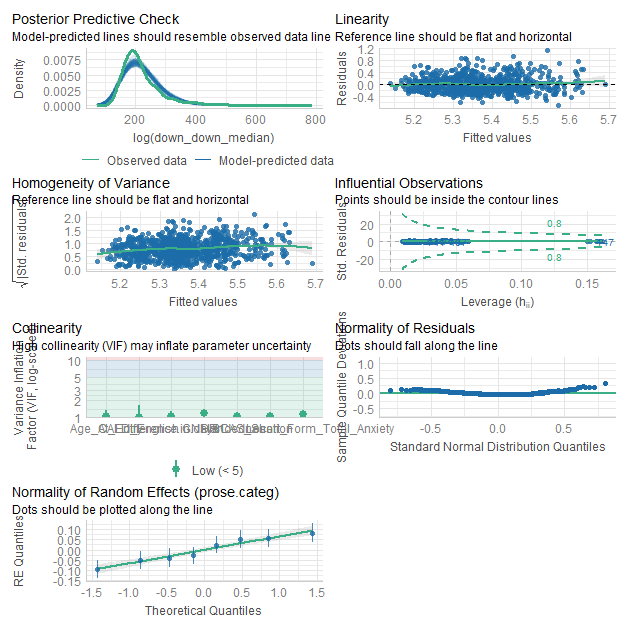

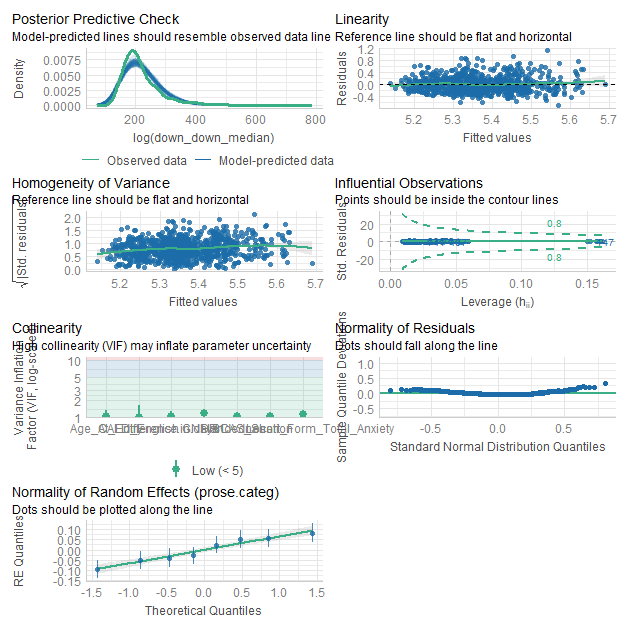


**
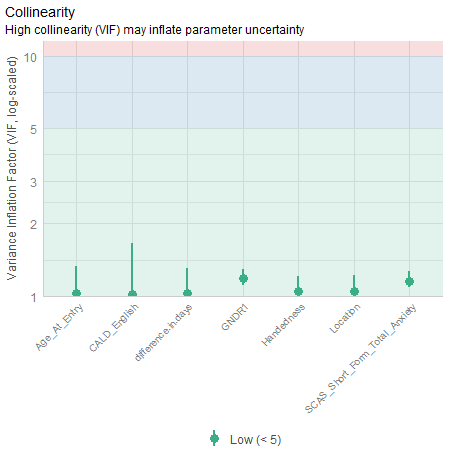
**

**Table 12. PHQ-A individual items and total keystrokes model**

|  | *Main Analysis* | | | | | | *Sensitivity Analysis* | | |
| --- | --- | --- | --- | --- | --- | --- | --- | --- | --- |
| Variable | Coefficient  (*b*) | Standard Error (SE) | 95% CI (Lower) | 95% CI (Upper) | *t* | *p* | Coefficient  (*b*) | *t* | *p* |
| Intercept \| Prose category (random effect)* | - | 7.91 | - | - | - | - | - | - | - |
| Intercept (fixed effect) | 65.14 | 28.50 | -2.43 | 132.72 | 2.29 | 0.057 | 60.98 | 2.52 | 0.012 |
| **PHQA - Item 1 (1^st^ degree)** | -76.54 | 27.2 | -141.03 | -12.05 | -2.81 | 0.026 | -88.92 | -2.67 | 0.008 |
| **PHQA - Item 1 (2^nd^ degree)** | -70.16 | 24.58 | -128.35 | -11.96 | -2.85 | 0.025 | -51.19 | -1.75 | 0.081 |
| PHQA - Item 2 | -1.77 | 1.08 | -4.34 | 0.80 | -1.63 | 0.147 | -1.72 | -1.03 | 0.305 |
| PHQA - Item 3 | 0.32 | 1.00 | -2.05 | 2.69 | 0.32 | 0.758 | 0.48 | 0.43 | 0.671 |
| PHQA - Item 4 | 0.18 | 0.69 | -1.46 | 1.83 | 0.26 | 0.799 | 0.48 | 0.39 | 0.696 |
| **PHQA - Item 5** | 4.54 | 0.87 | 2.46 | 6.62 | 5.19 | 0.001 | 4.19 | 3.31 | 0.001 |
| PHQA - Item 6 | -0.61 | 1.31 | -3.71 | 2.49 | -0.47 | 0.653 | 0.23 | 0.16 | 0.874 |
| PHQA - Item 7 | 0.43 | 0.86 | -1.60 | 2.46 | 0.51 | 0.629 | 0.12 | 0.1 | 0.923 |
| PHQA - Item 8 | 0.67 | 0.94 | -1.55 | 2.90 | 0.72 | 0.497 | 0.24 | 0.18 | 0.859 |
| PHQA - Item 9 | 1.08 | 1.19 | -1.78 | 3.94 | 0.91 | 0.397 | 0.66 | 0.4 | 0.690 |
| Handedness (Left hand) | -9.69 | 8.06 | -34.84 | 15.46 | -1.20 | 0.313 | -8.79 | -0.81 | 0.418 |
| Handedness (Not reported) | -12.25 | 1.86 | -16.64 | -7.85 | -6.60 | <.001 | -9.81 | -4.28 | <.001 |
| Handedness (Right hand) | -14.77 | 3.19 | -22.39 | -7.15 | -4.64 | 0.003 | -13.41 | -3.38 | 0.001 |
| Sex (Male) | -13.99 | 1.98 | -18.68 | -9.30 | -7.07 | <.001 | -14.15 | -6.7 | <.001 |
| Age | 2.26 | 1.85 | -2.12 | 6.64 | 1.22 | 0.261 | 2.26 | 1.31 | 0.189 |
| Location (Major Cities) | 7.51 | 2.48 | 1.62 | 13.39 | 3.03 | 0.020 | 8.2 | 3.55 | <.001 |
| Location (Outer Regional) | -7.59 | 3.36 | -15.98 | 0.80 | -2.26 | 0.068 | -7.17 | -1.25 | 0.213 |
| English spoken at home (True) | 0.62 | 3.02 | -6.68 | 7.92 | 0.20 | 0.845 | 2.93 | 0.75 | 0.453 |
| Days between assessment and typing task | 0.09 | 0.21 | -0.41 | 0.60 | 0.45 | 0.667 | 0.10 | 0.35 | 0.727 |

Note: *****SD is reported for random effect (not SE)

**Figure 10. Diagnostic Plots for PHQ-A individual items and total keystrokes model**


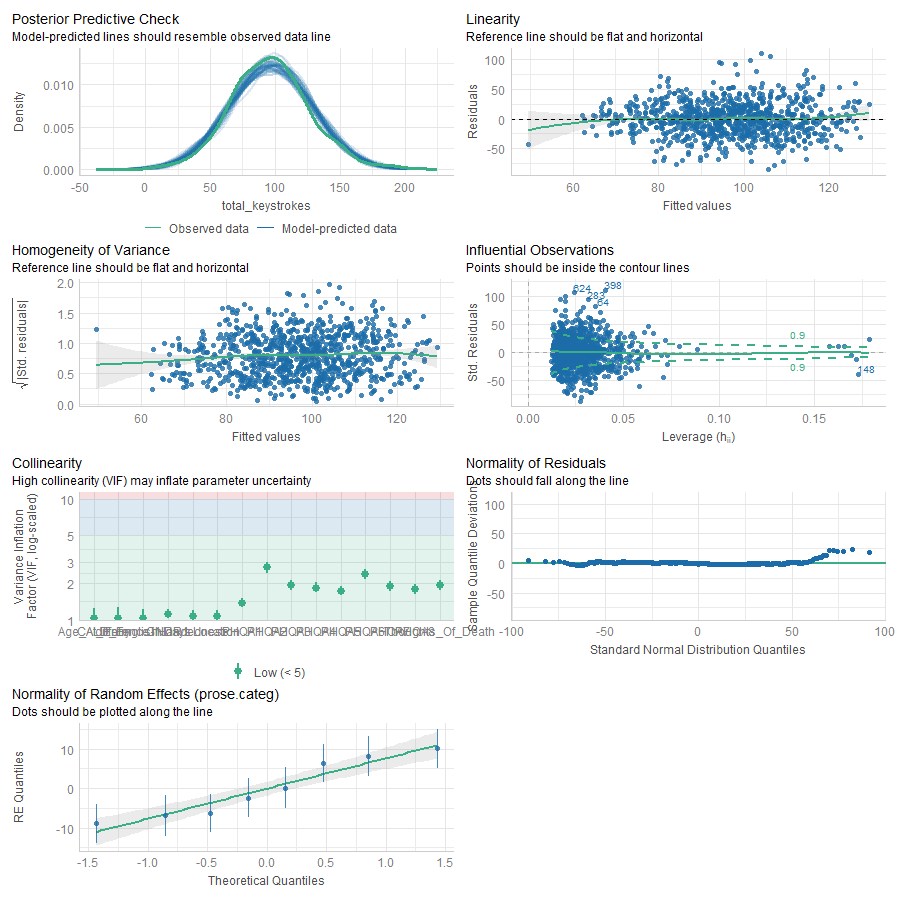

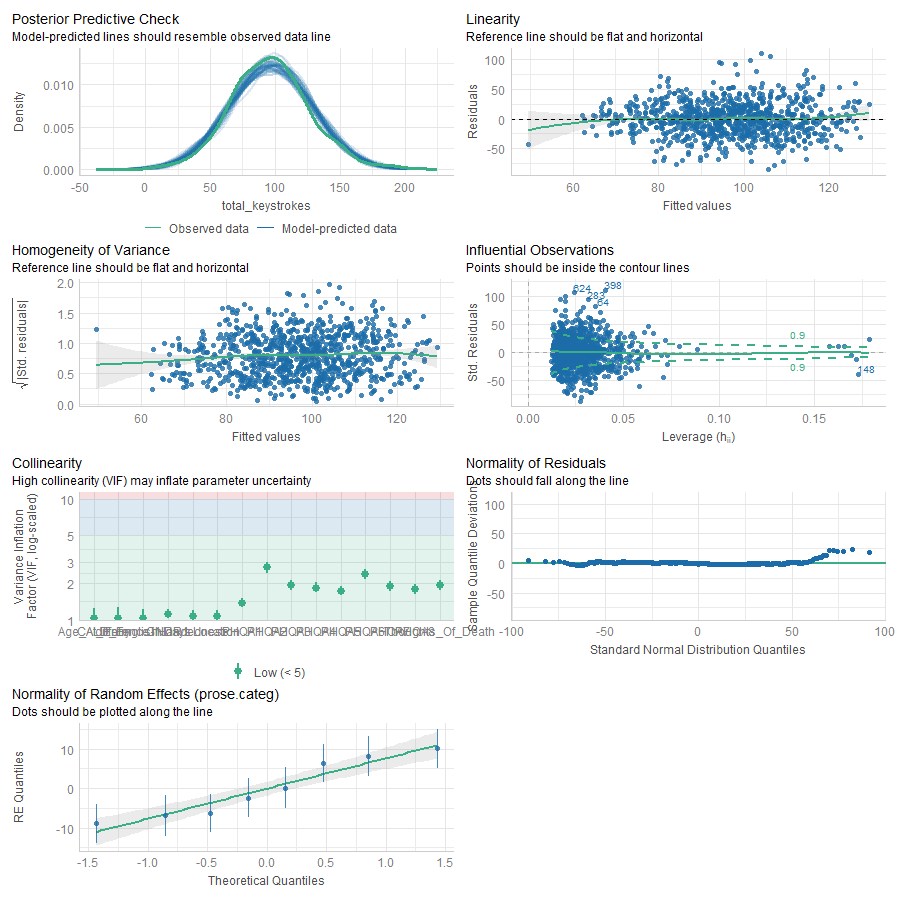


**
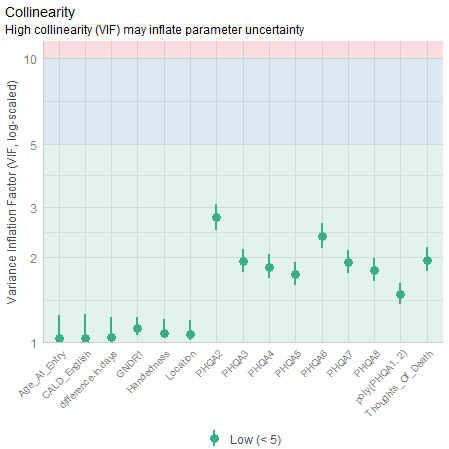
**

**Table 13. SDE total score and total keystrokes model**

|  | *Main Analysis* | | | | | | *Sensitivity Analysis* | | |
| --- | --- | --- | --- | --- | --- | --- | --- | --- | --- |
| Variable | Coefficient  (*b*) | Standard Error (SE) | 95% CI (Lower) | 95% CI (Upper) | *t* | *p* | Coefficient  (*b*) | *t* | *p* |
| Intercept \| Prose category (random effect)* | - | 8.12 | - | - | - | - | - | - | - |
| Intercept (fixed effect) | 70.21 | 25.52 | 9.62 | 130.81 | 2.75 | 0.029 | 66.72 | 2.79 | 0.005 |
| **SDE total score (1^st^ degree)** | 101.42 | 39.80 | 6.97 | 195.88 | 2.55 | 0.039 | 106.6 | 3.51 | <.001 |
| **SDE total score (2^nd^ degree)** | 50.34 | 20.31 | 1.82 | 98.87 | 2.48 | 0.044 | 57.14 | 2.00 | 0.045 |
| Handedness (Left hand) | -10.24 | 7.75 | -34.49 | 14.00 | -1.32 | 0.275 | -9.36 | -0.87 | 0.383 |
| Handedness (Not reported) | -12.44 | 1.69 | -16.45 | -8.44 | -7.37 | <.001 | -9.910 | -4.36 | <.001 |
| Handedness (Right hand) | -14.33 | 3.86 | -23.56 | -5.09 | -3.71 | 0.008 | -13.10 | -3.31 | 0.001 |
| Sex (Male) | -13.68 | 1.62 | -17.53 | -9.82 | -8.43 | <.001 | -13.38 | -6.11 | <.001 |
| Age | 2.06 | 1.69 | -1.96 | 6.08 | 1.22 | 0.263 | 2.03 | 1.19 | 0.235 |
| Location (Major Cities) | 7.49 | 2.47 | 1.63 | 13.36 | 3.03 | 0.019 | 8.05 | 3.49 | <.001 |
| Location (Outer Regional) | -8.2 | 3.68 | -17.41 | 1.00 | -2.23 | 0.071 | -7.15 | -1.26 | 0.206 |
| English spoken at home (True) | 1.46 | 2.83 | -5.36 | 8.29 | 0.52 | 0.622 | 3.80 | 0.99 | 0.325 |
| Days between assessment and typing task | 0.01 | 0.24 | -0.56 | 0.59 | 0.06 | 0.955 | 0.05 | 0.18 | 0.859 |

Note: *****SD is reported for random effect (not SE)

**Figure 11. SDE total score and total keystrokes model**


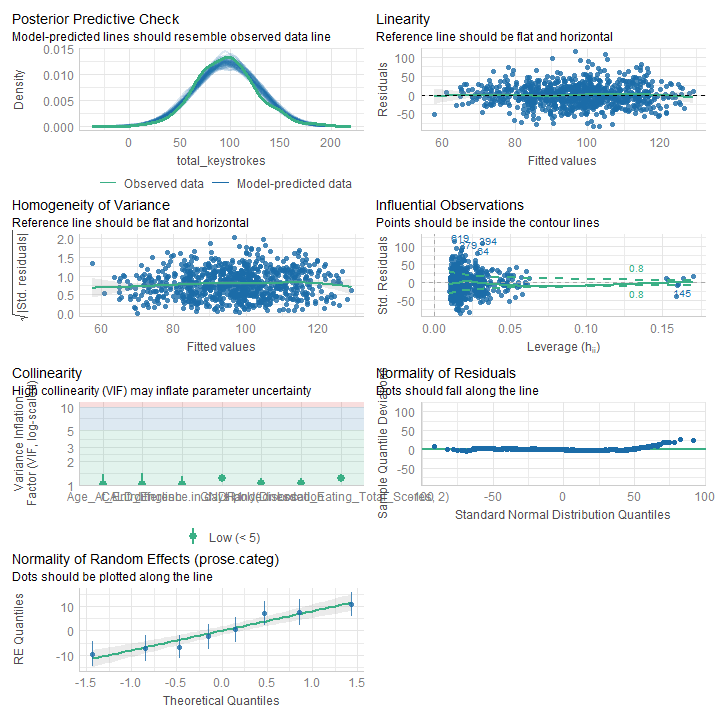

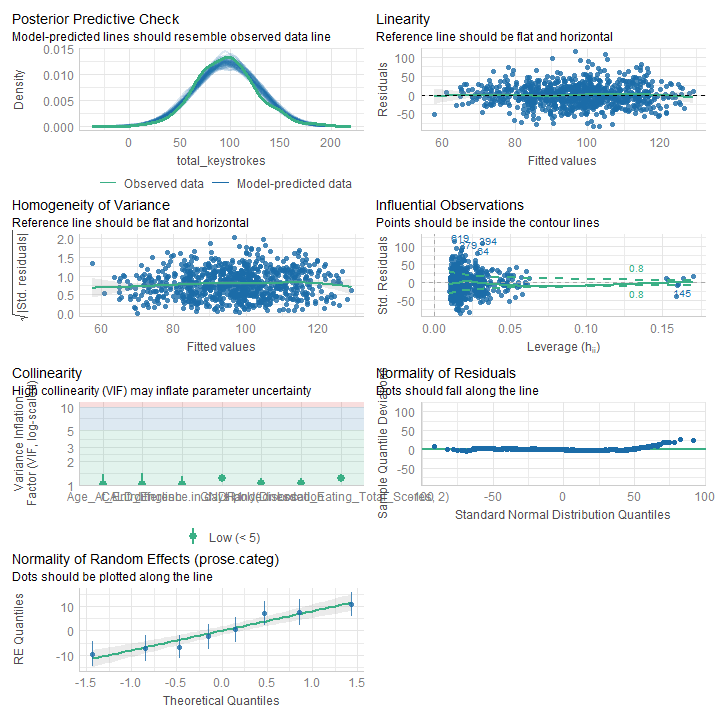


**
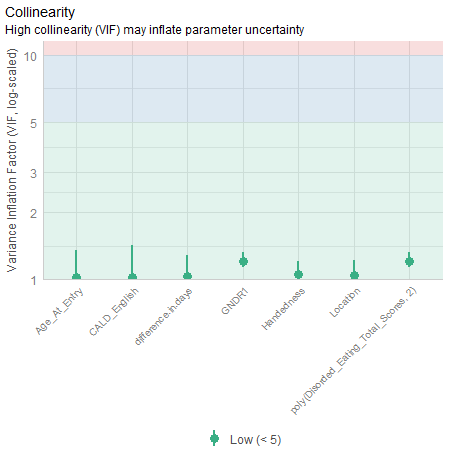
**

**Table 14. SDE positive screening and total keystrokes model**

|  | *Main Analysis* | | | | | | *Sensitivity Analysis* | | |
| --- | --- | --- | --- | --- | --- | --- | --- | --- | --- |
| Variable | Coefficient  (*b*) | Standard Error (SE) | 95% CI (Lower) | 95% CI (Upper) | *t* | *p* | Coefficient  (*b*) | *t* | *p* |
| Intercept \| Prose category (random effect)* | - | 7.87 | - | - | - | - | - | - | - |
| Intercept (fixed effect) | 64.68 | 24.74 | 5.94 | 123.43 | 2.61 | 0.035 | 59.71 | 2.49 | 0.013 |
| **SDE positive screening** | 6.22 | 2.13 | 1.18 | 11.26 | 2.92 | 0.023 | 6.76 | 3.34 | 0.001 |
| Handedness (Left hand) | -8.10 | 7.49 | -31.49 | 15.28 | -1.08 | 0.356 | -7.29 | -0.68 | 0.497 |
| Handedness (Not reported) | -12.24 | 1.63 | -16.10 | -8.39 | -7.53 | <.001 | -9.49 | -4.17 | <.001 |
| Handedness (Right hand) | -14.69 | 3.97 | -24.19 | -5.19 | -3.70 | 0.008 | -13.18 | -3.33 | 0.001 |
| Sex (Male) | -13.78 | 1.59 | -17.54 | -10.02 | -8.68 | <.001 | -13.38 | -6.24 | <.001 |
| Age | 2.21 | 1.66 | -1.74 | 6.15 | 1.33 | 0.226 | 2.24 | 1.31 | 0.190 |
| Location (Major Cities) | 7.94 | 2.50 | 2.00 | 13.88 | 3.17 | 0.016 | 8.62 | 3.74 | <.001 |
| Location (Outer Regional) | -7.52 | 3.57 | -16.46 | 1.42 | -2.10 | 0.084 | -6.75 | -1.19 | 0.234 |
| English spoken at home (True) | 1.55 | 2.91 | -5.47 | 8.57 | 0.53 | 0.612 | 3.95 | 1.02 | 0.306 |
| Days between assessment and typing task | 0.004 | 0.25 | -0.60 | 0.61 | 0.02 | 0.987 | 0.05 | 0.16 | 0.872 |

Note: *****SD is reported for random effect (not SE)

**Figure 12. SDE positive screening and total keystrokes model**


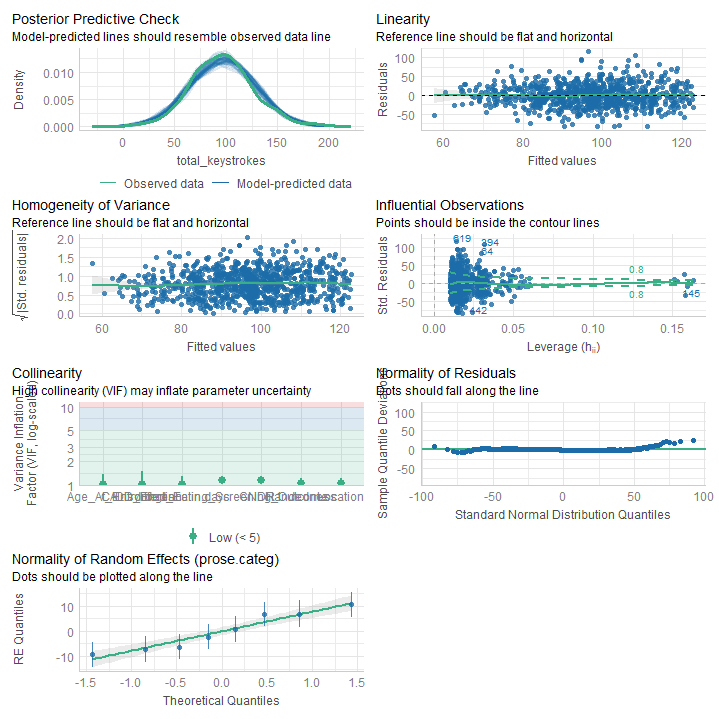

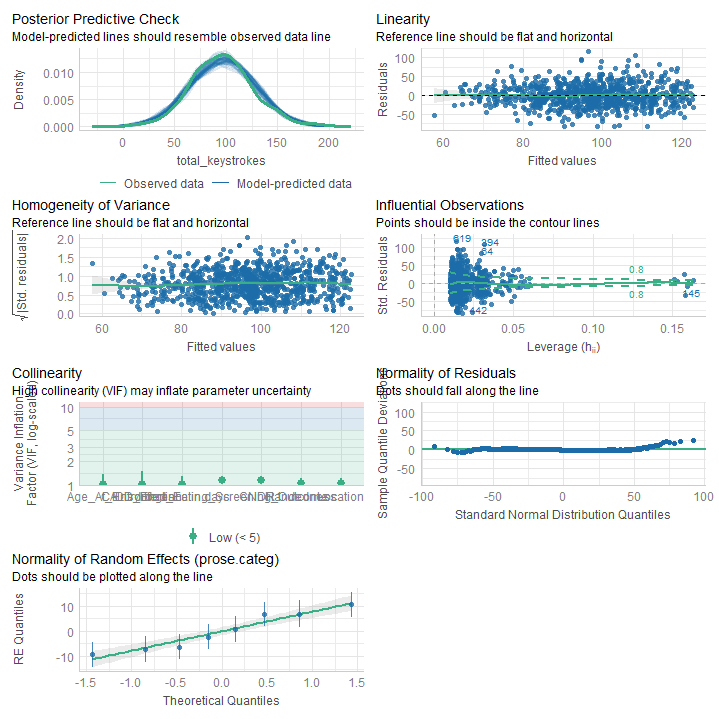


**
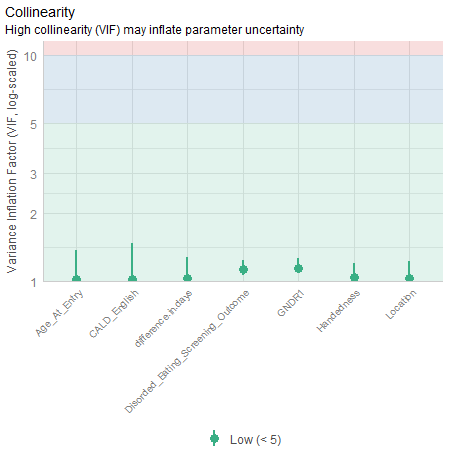
**

**Table 15. SDE individual items and total keystrokes model**

|  | *Main Analysis* | | | | | | *Sensitivity Analysis* | | |
| --- | --- | --- | --- | --- | --- | --- | --- | --- | --- |
| Variable | Coefficient  (*b*) | Standard Error (SE) | 95% CI (Lower) | 95% CI (Upper) | *t* | *p* | Coefficient  (*b*) | *t* | *p* |
| Intercept \| Prose category (random effect)* | - | 7.87 | - | - | - | - | - | - | - |
| Intercept (fixed effect) | 65.27 | 25.69 | 4.28 | 126.26 | 2.54 | 0.039 | 62.01 | 2.59 | 0.010 |
| SDE item 1 | 1.88 | 1.41 | -1.46 | 5.22 | 1.33 | 0.224 | 1.57 | 0.74 | 0.459 |
| SDE item 2 | 1.58 | 2.83 | -5.14 | 8.30 | 0.56 | 0.595 | 0.88 | 0.39 | 0.696 |
| SDE item 3 | 5.86 | 3.63 | -2.83 | 14.54 | 1.61 | 0.153 | 6.41 | 1.60 | 0.110 |
| SDE item 4 | 6.55 | 2.91 | -0.37 | 13.48 | 2.26 | 0.060 | 7.62 | 2.96 | 0.003 |
| SDE item 5 | -1.67 | 2.49 | -7.59 | 4.25 | -0.67 | 0.525 | -1.35 | -0.55 | 0.580 |
| Handedness (Left hand) | -9.98 | 6.88 | -31.27 | 11.31 | -1.45 | 0.238 | -9.28 | -0.86 | 0.388 |
| Handedness (Not reported) | -12.59 | 1.73 | -16.71 | -8.48 | -7.26 | <.001 | -9.79 | -4.30 | <.001 |
| Handedness (Right hand) | -14.88 | 3.73 | -23.8 | -5.97 | -3.99 | 0.006 | -13.57 | -3.43 | 0.001 |
| Sex (Male) | -12.89 | 1.32 | -16.01 | -9.77 | -9.80 | <.001 | -12.27 | -5.55 | <.001 |
| Age | 2.08 | 1.72 | -2.00 | 6.16 | 1.21 | 0.266 | 1.99 | 1.17 | 0.244 |
| Location (Major Cities) | 7.57 | 2.46 | 1.73 | 13.4 | 3.08 | 0.018 | 8.12 | 3.53 | <.001 |
| Location (Outer Regional) | -7.67 | 3.64 | -16.77 | 1.43 | -2.11 | 0.084 | -6.61 | -1.17 | 0.243 |
| English spoken at home (True) | 2.28 | 2.84 | -4.58 | 9.14 | 0.80 | 0.452 | 4.79 | 1.24 | 0.215 |
| Days between assessment and typing task | 0.02 | 0.25 | -0.57 | 0.61 | 0.08 | 0.935 | 0.05 | 0.15 | 0.879 |

Note: *****SD is reported for random effect (not SE)

**Figure 13. SDE individual items and total keystrokes model**


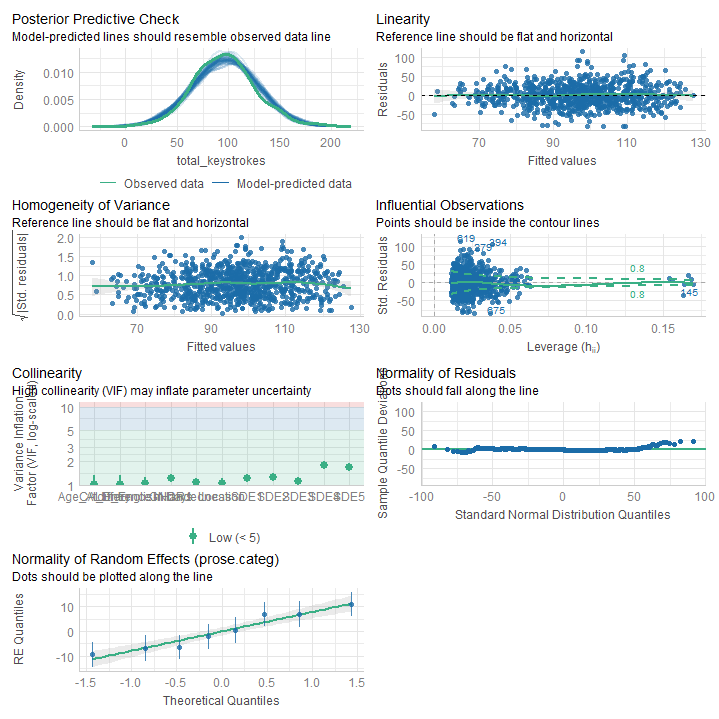

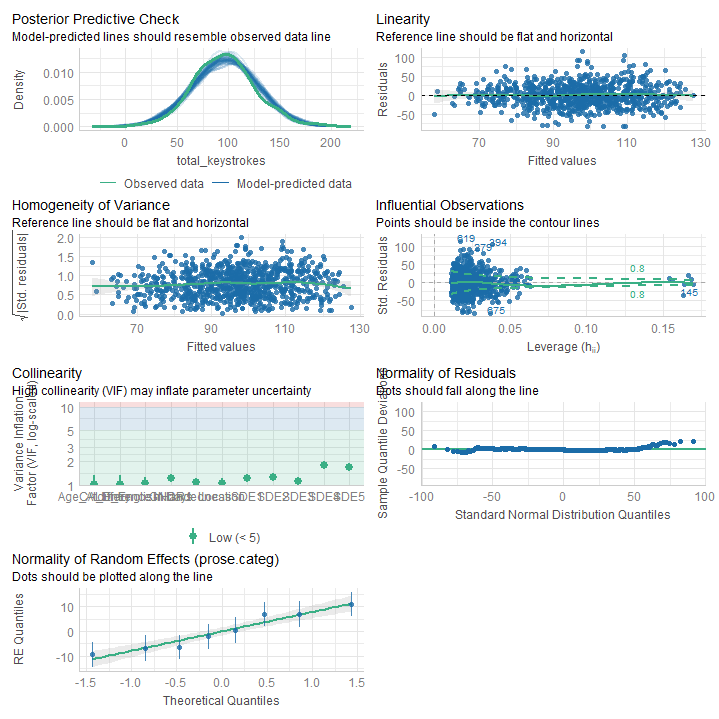


**
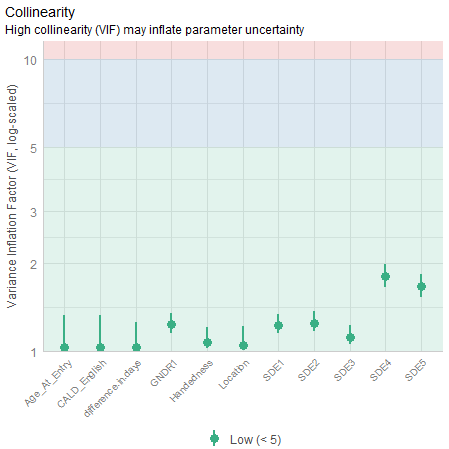
**

|  | *Main Analysis* | | | | | | *Sensitivity Analysis* | | |
| --- | --- | --- | --- | --- | --- | --- | --- | --- | --- |
| Variable | Coefficient  (*b*) | Standard Error (SE) | 95% CI (Lower) | 95% CI (Upper) | *t* | *p* | Coefficient  (*b*) | *t* | *p* |
| Intercept \| Prose category (random effect)* | - | 7.95 | - | - | - | - | - | - | - |
| Intercept (fixed effect) | 63.78 | 27.65 | -1.77 | 129.33 | 2.31 | 0.055 | 59.32 | 2.45 | 0.015 |
| ISI total score | 0.39 | 0.20 | -0.08 | 0.85 | 1.99 | 0.089 | 0.34 | 1.99 | 0.047 |
| Handedness (Left hand) | -9.29 | 8.30 | -35.2 | 16.62 | -1.12 | 0.342 | -8.37 | -0.77 | 0.442 |
| Handedness (Not reported) | -11.75 | 1.56 | -15.44 | -8.05 | -7.53 | <.001 | -9.10 | -3.97 | <.001 |
| Handedness (Right hand) | -14.34 | 3.66 | -23.08 | -5.60 | -3.92 | 0.006 | -12.94 | -3.25 | 0.001 |
| Sex (Male) | -14.51 | 2.05 | -19.37 | -9.66 | -7.09 | <.001 | -14.59 | -6.93 | <.001 |
| Age | 2.31 | 1.82 | -2.00 | 6.62 | 1.27 | 0.245 | 2.39 | 1.38 | 0.167 |
| Location (Major Cities) | 7.88 | 2.75 | 1.34 | 14.41 | 2.86 | 0.025 | 8.48 | 3.65 | <.001 |
| Location (Outer Regional) | -6.81 | 3.60 | -15.81 | 2.19 | -1.89 | 0.112 | -6.15 | -1.07 | 0.285 |
| English spoken at home (True) | 1.08 | 3.25 | -6.77 | 8.92 | 0.33 | 0.751 | 3.37 | 0.86 | 0.387 |
| Days between assessment and typing task | 0.12 | 0.22 | -0.4 | 0.64 | 0.54 | 0.608 | 0.15 | 0.5 | 0.617 |

**Table 16. ISI total score and total keystrokes model**

Note: *****SD is reported for random effect (not SE)

**Figure 14. ISI total score and total keystrokes model**


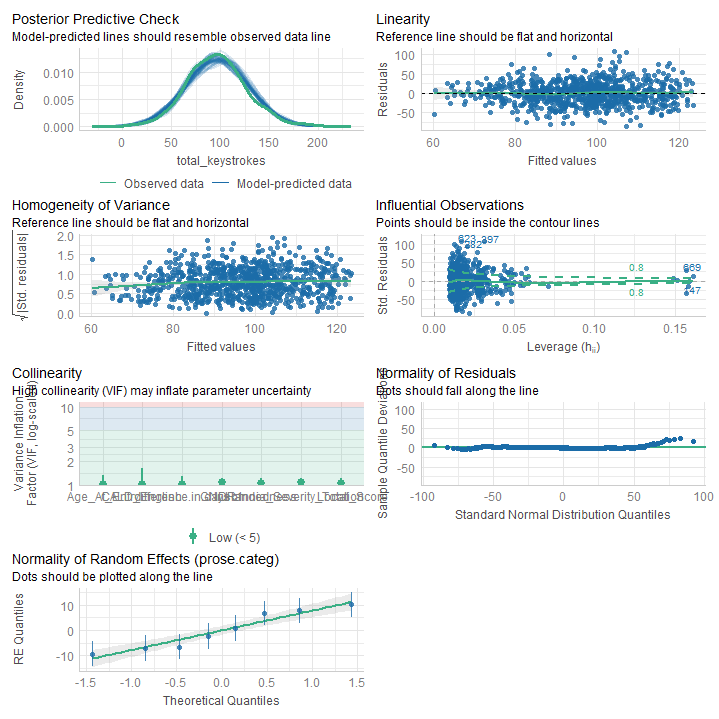

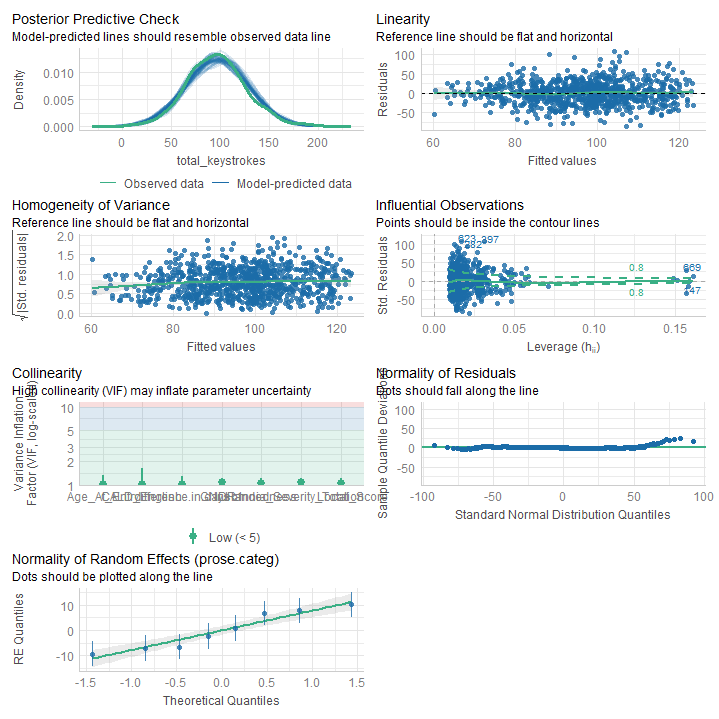


**
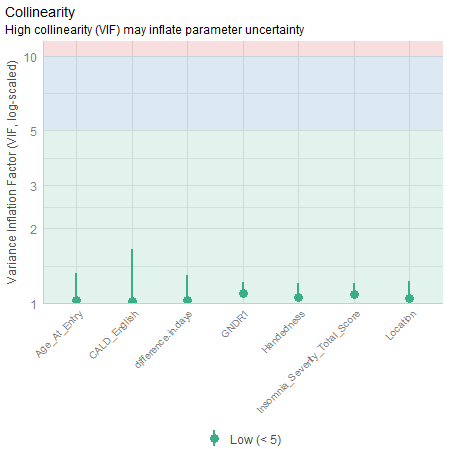
**

**Table 17. CAS-8 total score and total keystrokes model**

|  | *Main Analysis* | | | | | | *Sensitivity Analysis* | | |
| --- | --- | --- | --- | --- | --- | --- | --- | --- | --- |
| Variable | Coefficient  (*b*) | Standard Error (SE) | 95% CI (Lower) | 95% CI (Upper) | *t* | *p* | Coefficient  (*b*) | *t* | *p* |
| Intercept \| Prose category (random effect)* | - | 7.95 | - | - | - | - | - | - | - |
| Intercept (fixed effect) | 67.61 | 27.74 | 1.82 | 133.40 | 2.44 | 0.045 | 61.45 | 2.54 | 0.011 |
| CAS-8 total score | 0.12 | 0.17 | -0.28 | 0.52 | 0.73 | 0.491 | 0.13 | 0.69 | 0.491 |
| Handedness (Left hand) | -8.18 | 7.83 | -32.66 | 16.3 | -1.04 | 0.371 | -7.45 | -0.69 | 0.493 |
| Handedness (Not reported) | -11.87 | 1.58 | -15.62 | -8.13 | -7.51 | <.001 | -9.20 | -4.02 | <.001 |
| Handedness (Right hand) | -13.99 | 3.66 | -22.74 | -5.24 | -3.82 | 0.007 | -12.7 | -3.20 | 0.001 |
| Sex (Male) | -15.19 | 2.14 | -20.25 | -10.14 | -7.12 | <.001 | -15.06 | -6.90 | <.001 |
| Age | 2.19 | 1.85 | -2.20 | 6.58 | 1.18 | 0.276 | 2.36 | 1.37 | 0.172 |
| Location (Major Cities) | 7.88 | 2.73 | 1.40 | 14.36 | 2.89 | 0.024 | 8.48 | 3.65 | <.001 |
| Location (Outer Regional) | -7.35 | 3.72 | -16.64 | 1.94 | -1.98 | 0.100 | -6.67 | -1.16 | 0.245 |
| English spoken at home (True) | 0.98 | 3.16 | -6.65 | 8.61 | 0.31 | 0.767 | 3.21 | 0.82 | 0.410 |
| Days between assessment and typing task | 0.11 | 0.21 | -0.40 | 0.62 | 0.50 | 0.633 | 0.15 | 0.50 | 0.614 |

Note: *****SD is reported for random effect (not SE)

**Figure 15. CAS-8 total score and total keystrokes model**


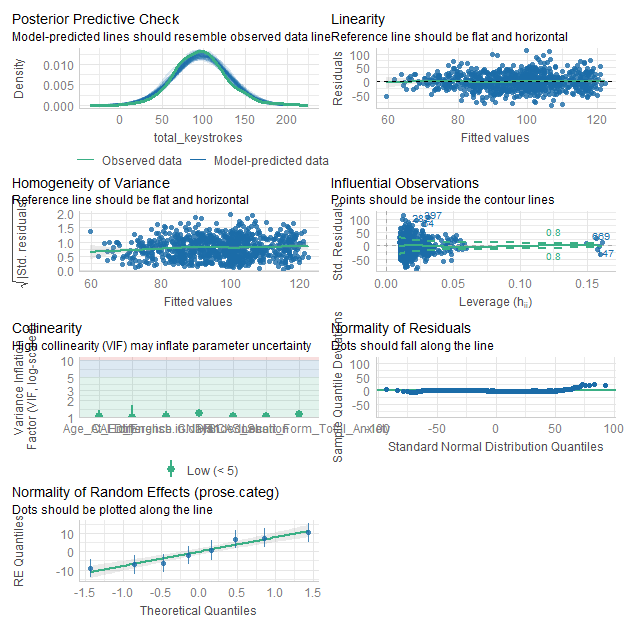

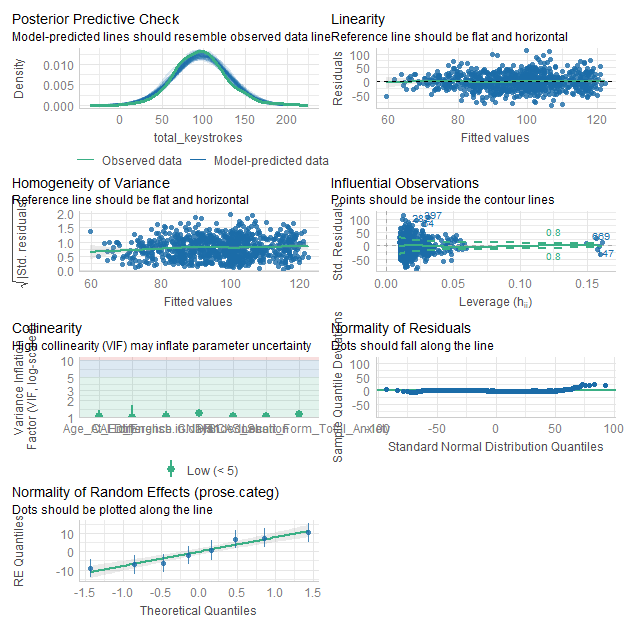


**
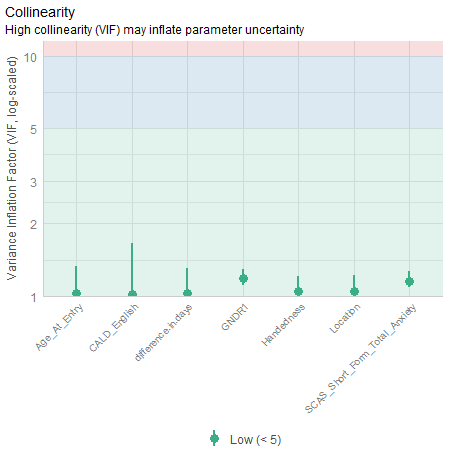
**
